# The IGF2BP3/Notch/Jag1 pathway: A key regulator of hepatic stellate cell ferroptosis in liver fibrosis

**DOI:** 10.1002/ctm2.1793

**Published:** 2024-08-07

**Authors:** Xinmiao Li, Yifei Li, Weizhi Zhang, Feng Jiang, Lifan Lin, Yining Wang, Lingling Wu, Han Zeng, Jianjian Zheng

**Affiliations:** ^1^ Zhejiang Key Laboratory of Intelligent Cancer Biomarker Discovery and Translation The First Affiliated Hospital of Wenzhou Medical University Wenzhou China; ^2^ School of Mental Health Wenzhou Medical University Wenzhou China; ^3^ Renji College Wenzhou Medical University Wenzhou China

**Keywords:** ferroptosis, IGF2BP3 deficiency, liver fibrosis, m^6^A methyltransferase

## Abstract

**Introduction:**

Liver fibrosis is primarily driven by the activation of hepatic stellate cells (HSCs), which involves various epigenetic modifications.

**Objectives:**

*N* ^6^‐methyladenosine (m^6^A), the most prevalent RNA modification in eukaryotic cells, influences numerous physiological and pathological processes. Nevertheless, the role of insulin‐like growth factor 2 mRNA‐binding protein 3 (IGF2BP3), a reader gene mediating m^6^A modifications, in liver fibrosis remains unclear.

**Methods and results:**

This study demonstrated that IGF2BP3 knockout reduces liver fibrosis by promoting HSC ferroptosis (FPT) and inactivating HSCs. Multi‐omics analysis revealed that HSC‐specific IGF2BP3 knockout decreased m^6^A content in Jagged1 (Jag1), a key component of the Notch signalling pathway. Furthermore, IGF2BP3 deficiency significantly reduced the expression of hairy and enhancer of split‐1 (Hes1), a transcription factor in the Notch/Jag1 signalling pathway, with mRNA levels declining to 35%–62% and protein levels to 28%–35%. Additionally, it suppressed glutathione peroxidase 4 (GPX4) (decreased to approximately 31%–38%), a negative regulator of FPT, thereby facilitating HSC FPT progression and reducing profibrotic gene expression.

**Conclusion:**

These findings uncover a novel IGF2BP3/Notch/Jag1 signalling pathway involving HSC FPT, suggesting promising targets for ameliorating liver fibrosis.

**Key Points/Highlights:**

- IGF2BP3 deficiency inactivates Jag1 signalling.
- IGF2BP3 deficiency‐mediated m^6^A modifications promote HSC ferroptosis.
- IGF2BP3 inhibition facilitates ferroptosis in HSCs via the Hes1/GPX4 axis.
- IGF2BP3 deficiency inactivates Jag1/Notch1/3/Hes1 signalling pathway inactivation, leading to the decrease in GPX4, which contributes to HSC ferroptosis.

## Graphical abstract

Schematic representation of the IGF2BP3 working model: HSC‐specific deletion of IGF2BP3 recognises Jag1 m^6^A binding sites, inhibiting the Notch pathway and inactivating the downstream molecule Hes1. This regulation of GPX4 ultimately leads to HSC FPT, alleviating liver fibrosis.

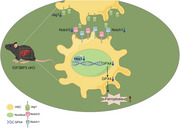

## 1 INTRODUCTION

Liver fibrosis, a pathological response to chronic liver injury, is marked by the activation of hepatic stellate cells (HSCs) and the deposition of extracellular matrix components, ultimately disrupting liver architecture.^1^ Without intervention, liver fibrosis inevitably progresses to cirrhosis and hepatocellular carcinoma (HCC). Activated HSCs are considered the principal drivers of liver fibrosis due to their excessive production of the extracellular matrix.^2^ Therefore, suppressing HSC activation is a critical objective in combating liver fibrosis.

The *N* ^6^‐methyladenosine (m^6^A) modification of RNA plays a vital regulatory role in the gene expression through three types of effector proteins: writers, readers and erasers.^3^ The methyl group is added to the adenosine base by a multi‐subunit complex (“writer”).^4^ This modification can be “read” by RNA‐binding proteins, primarily those from the YT521‐Bhomologydomain (YTH) family and the insulin‐like growth factor 2 mRNA‐binding proteins (IGF2BPs) family, and can be reversed by “eraser” proteins.^5^ Dysregulation of the “reader” IGF2BP3 affects m^6^A levels and disrupts various biological behaviors. For instance, in nasopharyngeal carcinoma, IGF2BP3 promotes proliferation and metastasis by altering m^6^A‐modified KPNA2 stability.^6^ IGF2BP3 also plays a role in the pathological and physiological regulation of HCC.^7^ However, its involvement in liver fibrosis remains unclear.

Ferroptosis (FPT) is a novel form of iron‐dependent cell death characterised by lipid peroxidation.^8^ Cells undergoing FPT exhibit distinctive shapes, biochemistry and genetics. Morphologically, FPT is identified by decreased mitochondrial volume, increased bilayer membrane density and the disappearance of mitochondrial cristae.^9^ Recent research has demonstrated a link between m^6^A modification and FPT in HCC.^10^ Consequently, we hypothesise that targeting m^6^A modification to induce FPT in HSCs could be an effective strategy for FPT‐based treatments.

This study explored the role of IGF2BP3 in liver fibrosis and uncovered a novel signalling pathway and molecular mechanisms of FPT in liver fibrosis. Our findings indicate that the m^6^A reader IGF2BP3 recognises Jag1 m^6^A binding sites to inhibit the Notch pathway and inactivate downstream molecule Hes1 to regulate GPX4, leading to HSC FPT. HSC‐specific deletion of IGF2BP3 enhanced HSC FPT and impeded their activation, thereby reducing liver fibrosis through the IGF2BP3/Notch/Jag1 signalling pathway. Thus, we identified IGF2BP3 as a novel regulator of liver fibrosis with significant translational potential for treating this condition.

## 2 MATERIALS AND METHODS

### 2.1 Animal experiments

*IGF2BP3^flox/flox^* mice and *Lrat‐Cre* mice were obtained from Cyagen Biosciences (Suzhou, China). *IGF2BP3^flox/flox^* mice were crossed with *Lrat‐Cre* mice to generate IGF2BP3 HSC‐specific knockout (cKO) mice, with *Lrat‐Cre* negative littermates serving as wild‐type controls.

Liver fibrosis was induced using carbon tetrachloride (CCl_4_), administered biweekly for 6 weeks. The control group (*n* = 6) received equivalent volumes of olive oil injections. To induce the non‐alcoholic steatohepatitis (NASH) model, mice (*n* = 6) were fed a high‐fat diet (HFD) for 16 weeks, whilst the control group (*n* = 6) was fed a normal diet. Post‐experiment, blood, liver tissue and primary HSC samples were collected for analysis.

### 2.1 Human cirrhosis biopsy specimens

This study involved collecting biopsy samples and sera from 30 patients with cirrhosis and 30 healthy individuals at the First Affiliated Hospital (FAH) of Wenzhou Medical University.

### 2.2 Serum alanine aminotransferase and aspartate aminotransferase assay

Serum levels of alanine aminotransferase (ALT) and aspartate aminotransferase (AST) in mice were measured using assay kits.

### 2.3 Quantitative real‐time PCR

Total RNA was extracted from primary HSCs, LX2 cells, and liver tissues using TRIzol reagents. The relative mRNA levels were quantified via the 2^−ΔΔCT^ method, with β‐actin as the internal control. Primer sequences for quantitative real‐time PCR (qRT‐PCR) are listed in Table S1.

### 2.4 Pathological evaluation of liver

Liver tissues were stained with hematoxylin and eosin (HE), Sirius Red, and Masson stains.^11^

### 2.5 Immunohistochemistry

Following previously described protocols,^12^ samples were fixed in formalin, blocked with 10% bovine serum albumin, incubated with antibodies. The stained regions were examined under a Carl Zeiss microscope. Antibody details are provided in Table S2.

### 2.6 Isolation and culture of primary hepatic stellate cells

Following previously described protocols,^13^ primary HSCs were isolated from control and IGF2BP3 cKO mice and the cells purity exceeding 98%.

### 2.7 Cell culture

LX2 cells, obtained from Newgainbio (Wuxi, China), were cultured in high‐glucose Dulbecco's modified eagle medium(DMEM) containing 10% Fetal Bovine Serum (FBS) at 37°C in an atmosphere with 5% CO_2_.

### 2.8 Cell viability assay

Cell viability was assessed using the Cell Counting Kit‐8 (CCK8) assay. In brief, 5 × 10^3^ primary HSCs and 8 × 10^3^ LX2 cells were seeded in 96‐well plates and subjected to the indicated treatments. Optical density at 450 nm was measured after 3 h using a microplate reader.

### 2.9 Western blot

Proteins were separated using 10% sodium dodecyl sulphate‐polyacrylamide gel electrophoresis and subsequently transferred to a polyvinylidene difluoride membrane and then incubated with antibodies. Details of the antibodies used are listed in Table S2.

### 2.10 Construction of cell lines with RNA knockdown and overexpression

Lentiviruses were constructed and packaged by *GenePharma* (Shanghai, China). Primary HSCs and LX2 cells were seeded in 24‐well plates for 24 h, achieving a cell fusion rate of 40%−60%. The lentivirus stock solution was diluted with 10% FBS to a concentration yielding the highest infection efficiency, determined to be 1:10. The puromycin concentration was maintained for over 120 h to select the optimal concentration for complete cell elimination, identified as 5 µg/mL. The resulting cells were collected for further analysis.

### 2.11 RNA‐sequencing profiling

Total RNA was isolated from primary HSCs of control and *IGF2BP3^−/−^* mice. Following quality assessment, RNA sequencing libraries were constructed and sequencing was performed using the Illumina NovaSeq 6000 platform. Differential expression analysis employed a threshold of |Log_2_ fold change| > .585 and adjusted *p* < .05.

### 2.12 m^6^A RNA immunoprecipitation‐sequencing

Primary HSCs were isolated and cultured in vitro for spontaneous activation. m^6^A RNA immunoprecipitation (RIP) was performed as previously described.^14^ An m^6^A antibody was used to enrich RNA fragments. Intact mRNA was isolated and fragmented purified mRNA into approximately 300‐nt fragments. Sequencing libraries were constructed and sequencing on the Illumina HiSeq 6000 platform.

### 2.13 mRNA m^6^A level

Purified mRNA was immobilised in assay wells, and total RNA was bound to strip wells. With the enhanced signal measured via absorbance using a microplate spectrophotometer, followed by colorimetric quantification.

### 2.14 Boron‐dipyrromethene staining

Primary HSCs were cultured in vitro for 1, 3, or 7 days and stained with boron‐dipyrromethene (BODIPY) for 30 min before undergoing BODIPY staining.^15^

### 2.15 Immunofluorescence

Liver tissues were fixed, embedded and sectioned into 3‐µm‐thick slices. Primary HSCs and LX2 cells were fixed, permeabilised, blocked, and incubated with antibodies. Details of the antibodies used are provided in Table S2.

### 2.16 Transmission electron microscopy

Cells were fixed, embedded, dehydrated and treated with uranyl acetate and lead citrate. Representative images were captured using transmission electron microscope (TEM).

### 2.17 Iron measurements

As previously described,^16^ an acidic buffer was added to the iron assay buffer to liberate the iron. After removing insoluble material, a microplate fluorometer determined the iron concentration.

### 2.18 Lipid reactive oxygen species assay

As previously described,^16^ cells were treated with Ferrostatin‐1 (Fer‐1) or RAS‐selective lethal 3 (RSL3) for 24 h, followed by incubation with BODIPY‐C11 dye and the amount of reactive oxygen species (ROS) was measured.

### 2.19 Detection of malondialdehyde and glutathione

Malondialdehyde (MDA) and glutathione (GSH) levels were quantified using activity assay kits. The contents of MDA and GSH were measured at 450 and 405 nm, respectively, using a microplate fluorometer.

### 2.20 Chromatin immunoprecipitation

GENMED's chromatin immunoprecipitation (ChIP) Enzymatic Chromatin IP kit was employed for ChIP. Cells were lysed with SDS buffer, and DNA was fragmented into 100−500 bp pieces via ultrasound. DNA fragments were then immunoprecipitated using specific antibodies or normal mouse IgG, and the enriched sequences were analysed by qRT‐PCR.

### 2.21 Luciferase assay

The luciferase activity was measured as previously described.^17^ GPX4‐WT and GPX4‐MUT plasmids were co‐transfected with Hes1 into HEK293 cells, and after 48 h, relative luciferase activity was assessed.

### 2.22 Statistical analysis

Statistical analyses were conducted using Prism. All data underwent a normality test in Prism. For data with a normal distribution, Student's *t*‐test and one‐way analysis of variance were used, whilst the Mann–Whitney test was applied for non‐normal data. Results were expressed as mean ± standard deviation (SD), with statistical significance determined at *p* < .05.

## 3 RESULTS

### 3.1 Hepatic stellate cell‐specific deficiency of IGF2BP3 attenuates liver fibrosis in mice

**FIGURE 1:**
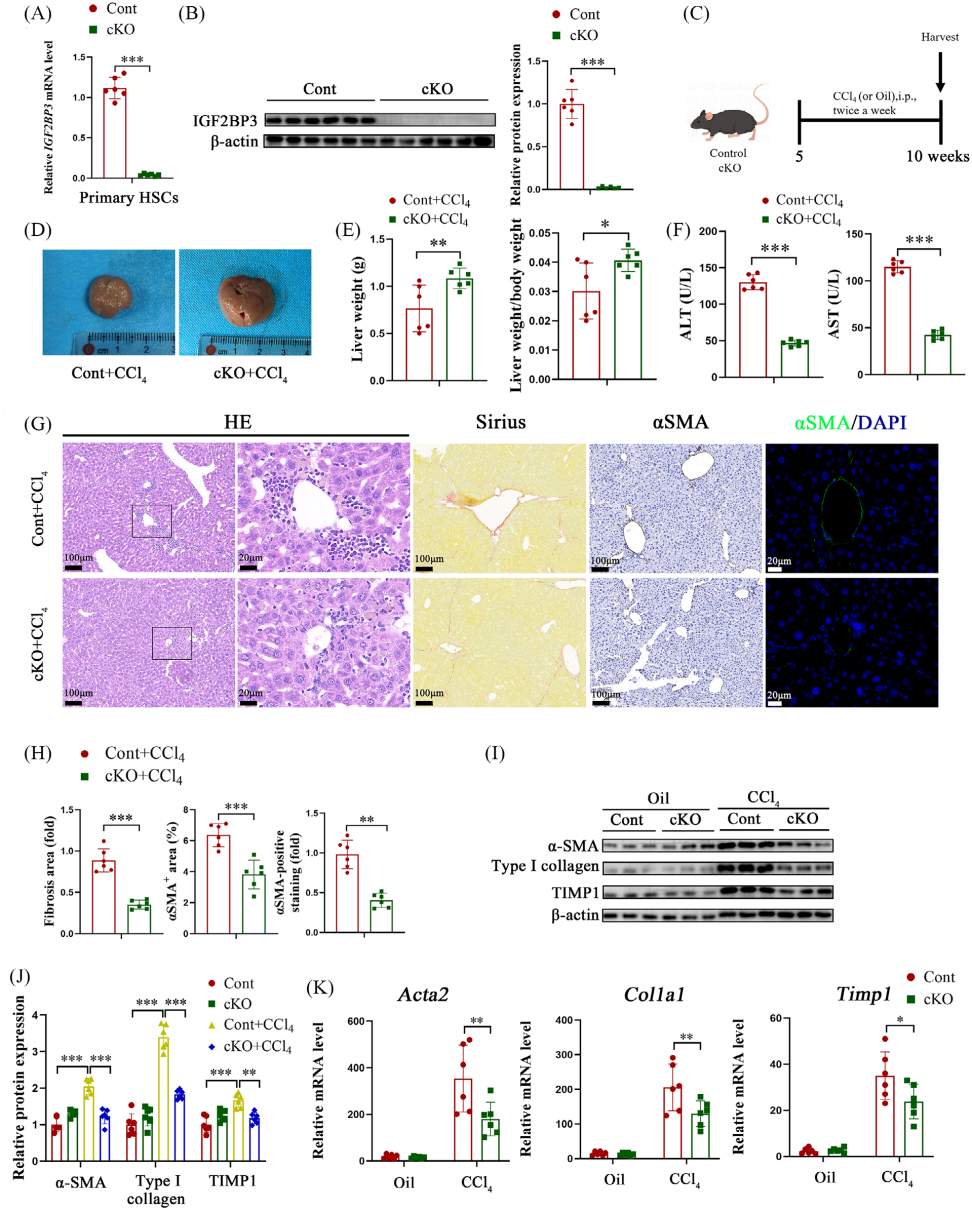
HSC‐specific knockout of IGF2BP3 alleviates CCl_4_‐induced liver fibrosis. Primary HSCs liver, and serum were isolated from control and IGF2BP3 cKO mice. (A) qRT‐PCR for IGF2BP3 in primary HSCs. (B) Western blot for IGF2BP3 in primary HSCs. (C) Illustration of the CCl_4_ experimental design. (D,E) Liver weight and liver weight to body weight ratio, with representative gross appearance of livers (D). (F) Levels of ALT and AST. (G,H) HE staining, Sirius red staining, and immunohistochemical and immunofluorescence staining for α‐SMA. Cell nuclei were stained with DAPI (blue) in immunofluorescence staining, as well as in subsequent similar experiments. (I–K) Expression of pro‐fibrosis markers in primary HSCs. Data are presented as mean ± SD from six experiments. *p < .05, **p < .01, ***p < .001. HSC, hepatic stellate cell; ALT, alanine aminotransferase; AST, aspartate aminotransferase; SD, standard deviation.

**FIGURE 2:**
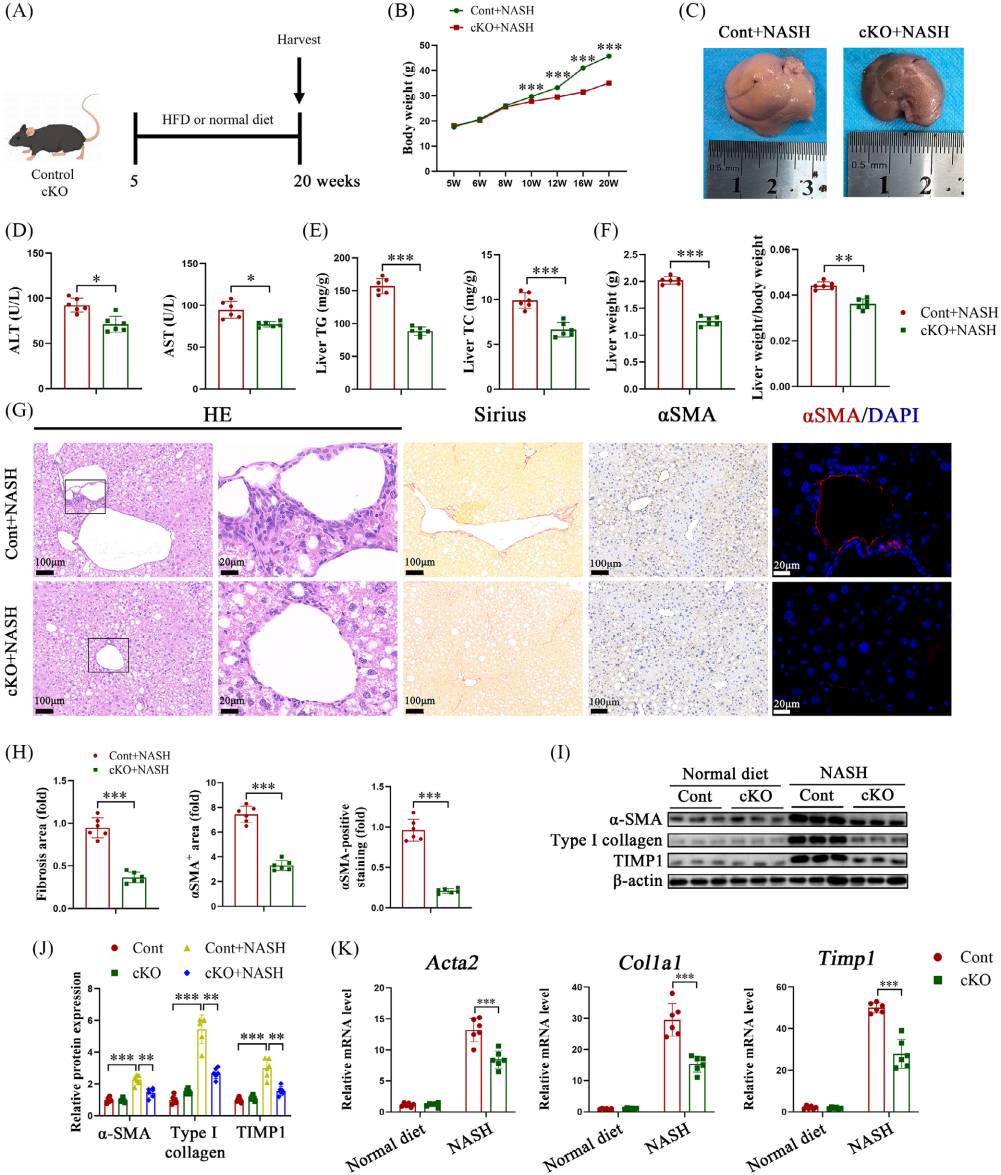
IGF2BP3 deficiency inhibits NASH‐induced liver fibrosis. Primary HSCs, liver and serum were isolated from control and IGF2BP3 cKO mice. (A) Experimental design of NASH‐induced liver fibrosis. (B) Body weight. (C) Representative gross appearance of livers. (D) Levels of ALT and AST. (E) Liver Triglyceride (TG) and Total Cholesterol (TC) content. (F) Liver weight and liver weight to body weight ratio. (G,H) HE staining, Sirius red staining, and immunohistochemical and immunofluorescence staining for α‐SMA. (I–K) Expression of pro‐fibrosis markers in primary HSCs. Data are presented as mean ± SD from six experiments. *p < .05, **p < .01, ***p < .001. NASH, Non‐alcoholic steatohepatitis; HSC, hepatic stellate cell; ALT, alanine aminotransferase; AST, aspartate aminotransferase; SD, standard deviation.

IGF2BP3 cKO mice were utilised to investigate the role of IGF2BP3 in liver fibrosis (Figures 1C and 2A). Primary HSCs from these cKO mice were examined to assess the silencing efficiency of IGF2BP3 (Figure 1A,B). When fed a normal diet, the cKO mice exhibited no discernible abnormalities, and liver pathology and function were comparable to control mice (Figure S1A–S1E).

To determine the impact of IGF2BP3 deletion in HSCs on liver fibrosis progression, liver fibrosis models were established using CCl_4_ and NASH. IGF2BP3 cKO mice treated with CCl_4_ showed ameliorated liver fibrosis compared to their control littermates. IGF2BP3 knockout resulted in increased body and liver weight and reduced ALT/AST levels and collagen content (Figure 1D–K, Figure S1B). Additionally, IGF2BP3 cKO mice exhibited decreased levels of α‐SMA and tissue inhibitor of metalloproteinases‐1 (Timp1) (Figure 1G–K). Similarly, IGF2BP3 knockout inhibited liver fibrosis in the NASH mouse model (Figure 2). In conclusion, HSC‐specific silencing of IGF2BP3 mitigated liver fibrosis in vivo.

### 3.2 Silencing IGF2BP3 induces hepatic stellate cell inactivation

**FIGURE 3:**
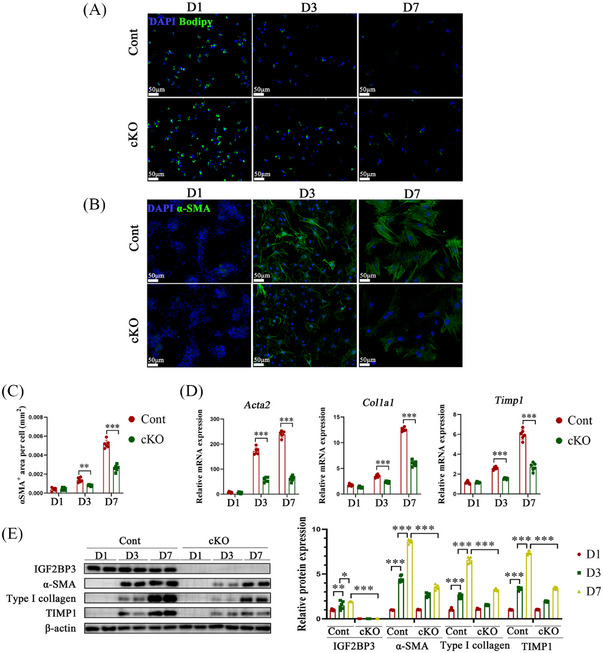
IGF2BP3 deficiency hinders the activation of primary HSCs and the expression of pro‐fibrosis genes. (A) Primary HSCs were isolated from control and IGF2BP3 cKO mice and cultured in vitro for 1, 3 and 7 days. (A) Fluorescent photographs of BODIPY staining. (B,C) Immunofluorescence staining for α‐SMA. (D,E) Expression of pro‐fibrosis markers in primary HSCs. Data are presented as mean ± SD from three experiments. *p < .05, **p < .01, ***p < .001. HSC, Hepatic stellate cell; BODIPY, boron‐dipyrromethene; SD, standard deviation.

**FIGURE 4:**
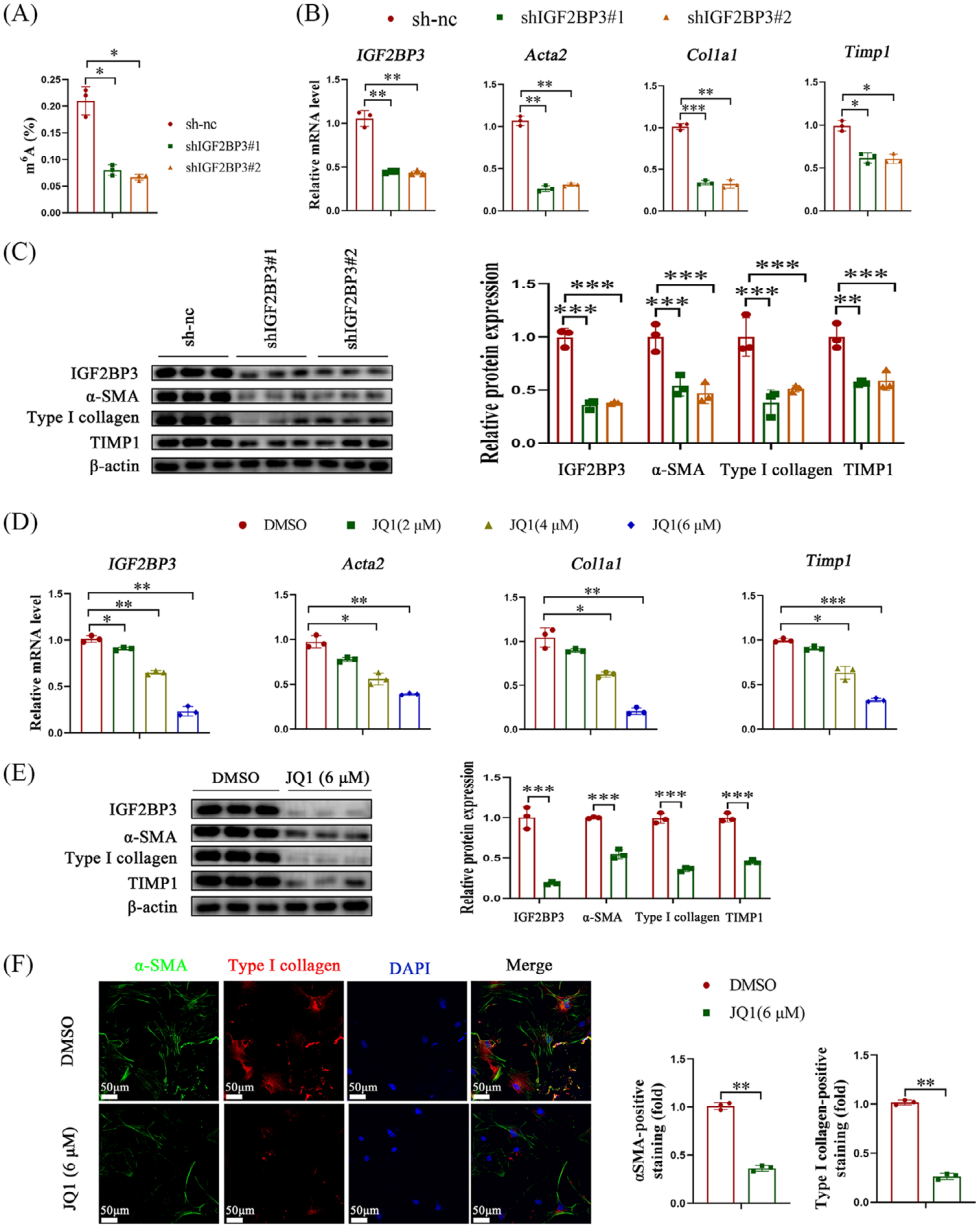
Inhibition of IGF2BP3 blocks the activation and expression of pro‐fibrosis markers in primary HSCs. Primary HSCs isolated from control mice were stably transfected with IGF2BP3 shRNA or treated with JQ1 for 24 h. (A) m^6^A levels. (B,C) Levels of IGF2BP3 and pro‐fibrosis markers in HSCs with IGF2BP3 shRNA. (D,E) Levels of IGF2BP3 and pro‐fibrosis markers in HSCs with JQ1 treatment. (F) Immunofluorescence staining of α‐SMA and Type I collagen. Data are presented as mean ± SD from three experiments. HSC, Hepatic stellate cell; SD, standard deviation.

To examine the impact of IGF2BP3 cKO on HSC activation status, primary HSCs were isolated from IGF2BP3 cKO mice. Compared to HSCs from control mice, those from IGF2BP3 cKO mice displayed a reduced rate of retinyl ester‐positive intracytoplasmic lipid droplet loss (Figure 3A). Additionally, the levels of α‐SMA, collagen type 1 alpha 1 (Col1A1), and Timp1, markers of HSC activation, were diminished (Figure 3B–E). Short hairpin RNAs were utilised to evaluate the effect of IGF2BP3 knockout on m^6^A levels and HSC inactivation. A significant reduction in the m^6^A content was observed in IGF2BP3‐deficient HSCs (Figure 4A and Figure S2A). HSC activation markers were downregulated in IGF2BP3‐lacking HSCs (Figure 4B,C, Figure S2B and S2C). Furthermore, treatment with JQ1, a bromodomain and extraterminal domain inhibitor (BETi) of IGF2BP3, significantly attenuated HSC activation markers (Figure 4D–F and Figure S2D–S2F). These results indicate that inhibiting IGF2BP3 suppresses HSC activation.

### 3.3 IGF2BP3 inhibition facilitates ferroptosis of hepatic stellate cells by suppressing GPX4

**FIGURE 5:**
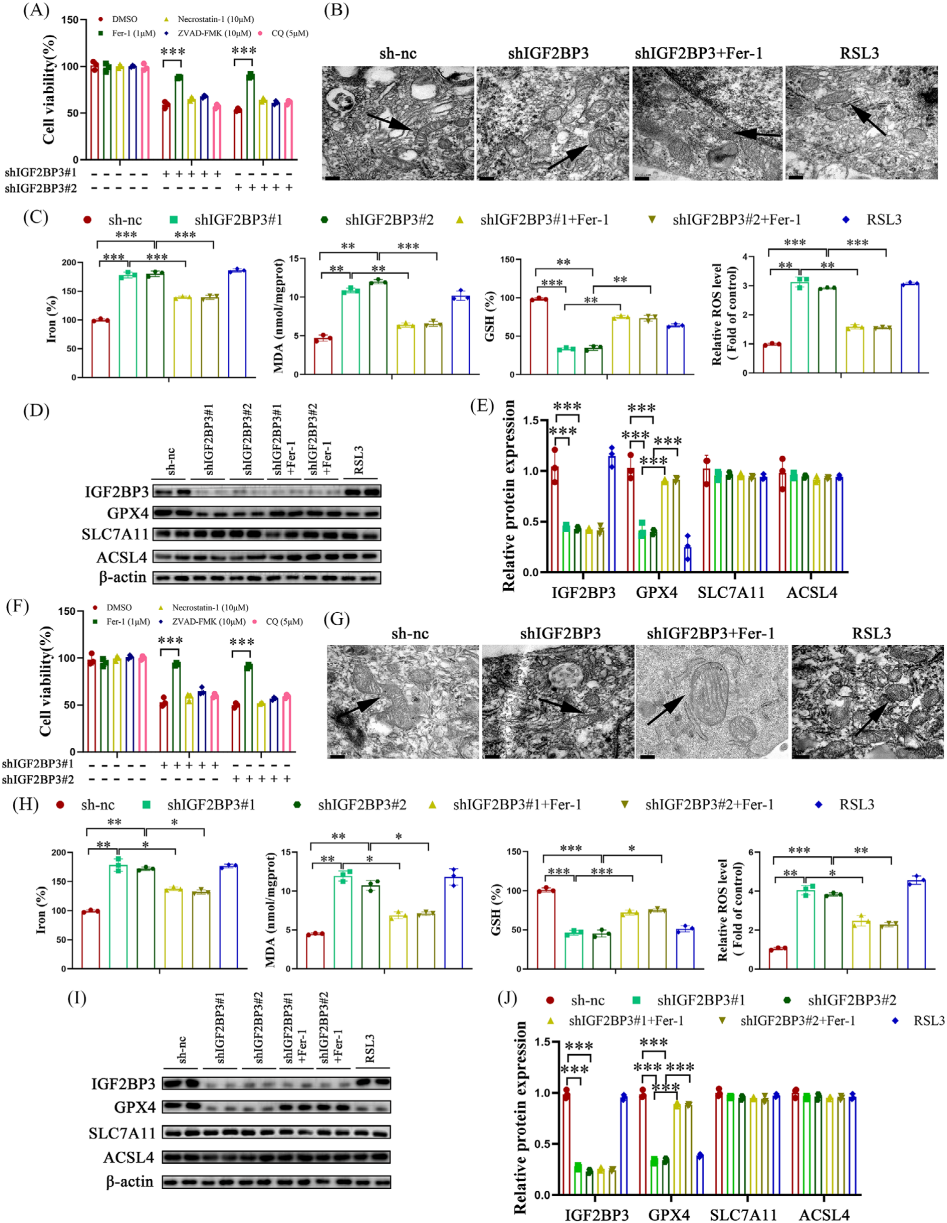
IGF2BP3 knockdown enhances HSC FPT via GPX4. Cells were transfected with IGF2BP3 shRNA and then treated with Fer‐1 (1 µM), Necrostatin‐1 (10 µM), ZVAD‐FMK (10 µM), CQ (5 µM), or RSL3 (1 µM) for 24 h. (A) Cell viability in LX2 cells. (B) Transmission electron microscopy (TEM). Scale bar,.2 µm. (C) Measurement of iron, MDA, GSH, and ROS levels in LX2 cells. (D,E) Protein expression of FPT‐related genes in LX2 cells. (F) Cell viability in primary HSCs. (G) TEM. (H) Measurement of iron, MDA, GSH, and ROS levels in primary HSCs. (I,J) Protein expression of FPT‐related genes in primary HSCs. Data are presented as mean ± SD from three experiments. *p < .05, **p < .01, ***p < .001. HSC, Hepatic stellate cell; MDA, malondialdehyde; GSH, glutathione; ROS, reactive oxygen species; FPT, ferroptosis; SD, standard deviation.

Recent studies have demonstrated an association between IGF2BP3 and cell FPT.^7^ Therefore, the effect of IGF2BP3 inhibition on HSC FPT was explored. Loss of IGF2BP3 in LX2 cells increased cell death, which was reversed by ferrostatin‐1 (Fer‐1), an FPT inhibitor (Figure 5A). Cell death was not influenced by Z‐VAD‐fluoromethylketone (apoptosis inhibitor), necrostatin‐1 (necroptosis inhibitor), or chloroquine (CQ), an autophagy inhibitor, similar to findings in primary HSCs (Figure 5F). These results demonstrate FPT involvement in IGF2BP3‐mediated HSC activation. Additionally, IGF2BP3 knockdown or treatment with RSL3, an FPT inducer, disrupted mitochondrial cristae, a process reversed by Fer‐1 (Figure 5B). IGF2BP3‐deficient cells exhibited FPT indicators, including elevated levels of iron, MDA, and ROS, along with depleted GSH, all of which were inhibited by Fer‐1 (Figure 5C). Inhibition of IGF2BP3 significantly promoted FPT in primary HSCs (Figure 5G,H). Key FPT markers, such as GPX4, solute carrier family 7 member 11 (SLC7A11), and acyl‐CoA synthetase long‐chain family member 4 (ACSL4), are involved in FPT progression. GPX4 levels, which negatively regulate FPT, were reduced in IGF2BP3‐deficient LX2 cells and primary HSCs (Figure 5D,E,I,J), whilst the expression of SLC7A11 and ACSL4 remained unaffected by IGF2BP3 inhibition.

In vivo, IGF2BP3 cKO mice exhibited higher levels of FPT indicators, such as iron, MDA, ROS, and depleted GSH, compared to NASH or CCl_4_ model control mice. These levels were inhibited by Fer‐1 (Figure S3A,D). Fer‐1 also prevented the IGF2BP3 cKO‐induced reduction in collagen levels (Figure S3B and S3E). Consistent with in vitro findings, GPX4 was downregulated in IGF2BP3 cKO mice (Figure S3,F). Collectively, these results suggest that inhibiting IGF2BP3 facilitates HSC FPT, at least in part, by suppressing GPX4.

### 3.4 IGF2BP3 deficiency‐mediated m^6^A modification inactivates Jag1 signalling

**FIGURE 6:**
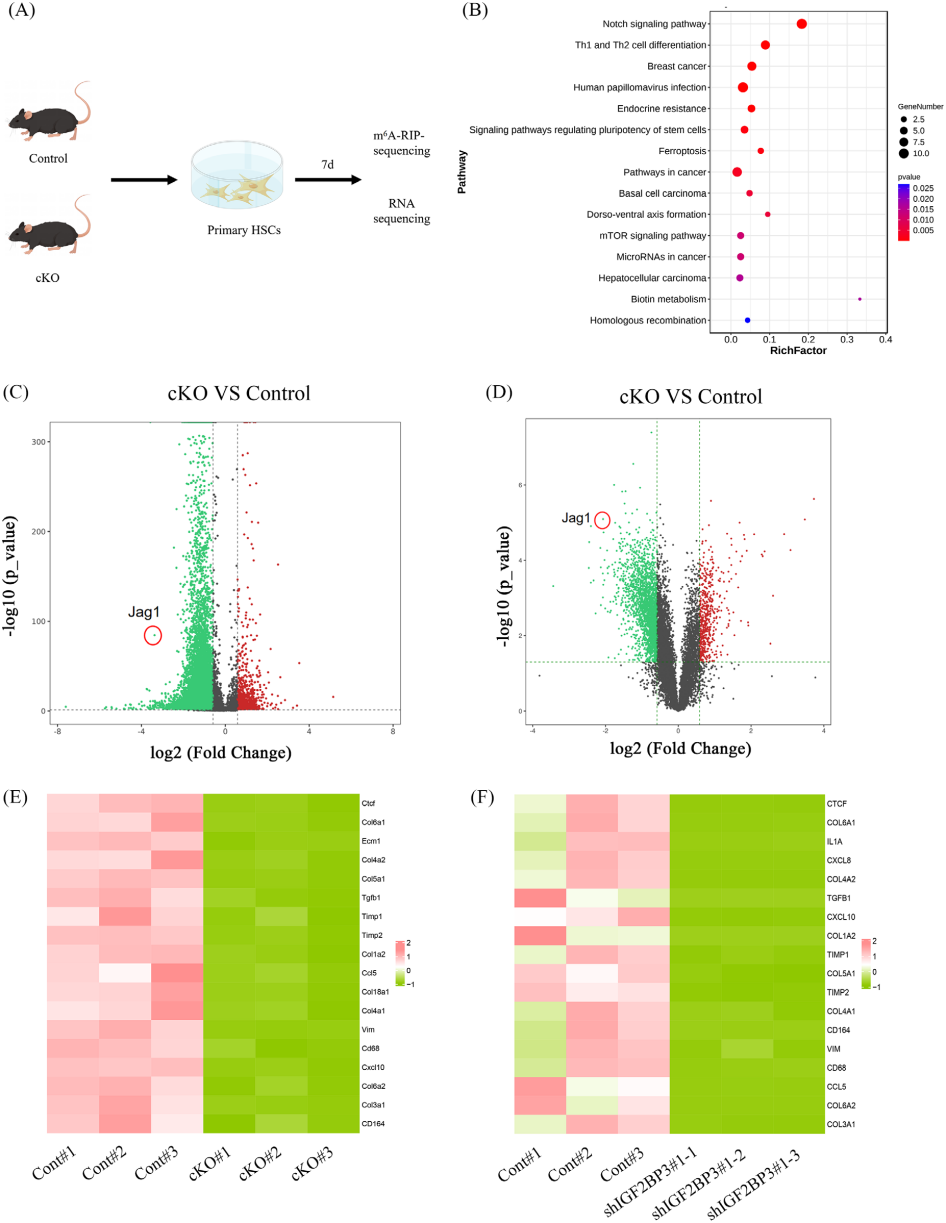
IGF2BP3‐mediated m^6^A modification regulates liver fibrosis by controlling Notch/Jag1 signalling. Primary HSCs were isolated from control and cKO mice and cultured in vitro for spontaneous activation. (A) m^6^A‐RIP sequencing and RNA sequencing scheme. (B) Gene set enrichment analysis (GSEA) identifying Kyoto Encyclopedia of Genes and Genomes (KEGG) pathways associated with differentially methylated genes. (C) Methylation differences between control HSCs and IGF2BP3 cKO HSCs; peaks in green indicate hypomethylation, and peaks in red indicate hypermethylation. (D) Differential expression analysis between control HSCs and IGF2BP3 cKO HSCs; genes in green indicate low expression, and genes in red indicate high expression. (E) Heatmap of classical pro‐fibrotic gene expression levels in primary HSCs. (F) Heatmap of classical pro‐fibrotic gene expression levels in LX2 cells. HSC, Hepatic stellate cell.

To elucidate the mechanism by which IGF2BP3 deficiency mitigates liver fibrosis and suppresses HSC activation, m^6^A RIP‐seq was conducted on isolated primary HSCs (Figure 6A). Downregulated genes in HSCs from IGF2BP3 cKO mice were predominantly enriched in the Notch pathway (Figure 6B). IGF2BP3 inhibition resulted in 16,949 hypomethylated sites and 961 hypermethylated sites (Figure 6C), highlighting IGF2BP3's critical role in HSC activation. Notably, Jag1, a hypomethylated transcript due to IGF2BP3 knockdown, emerged as the most downregulated gene in the Notch pathway (Figure 6C). Transcriptome analysis corroborated that Jag1 was significantly downregulated in the Notch pathway (Figure 6D). Thus, Notch‐Jag1 signalling appears to mediate the effects of IGF2BP3‐induced m^6^A modifications on HSC activation. Differentially expressed genes between the control and IGF2BP3 cKO groups were analysed to explore IGF2BP3's regulatory role, revealing downregulation of pro‐fibrosis‐related genes (Figure 6E). Similar results were observed in LX2 cells following IGF2BP3 inhibition (Figure 6F).

**FIGURE 7:**
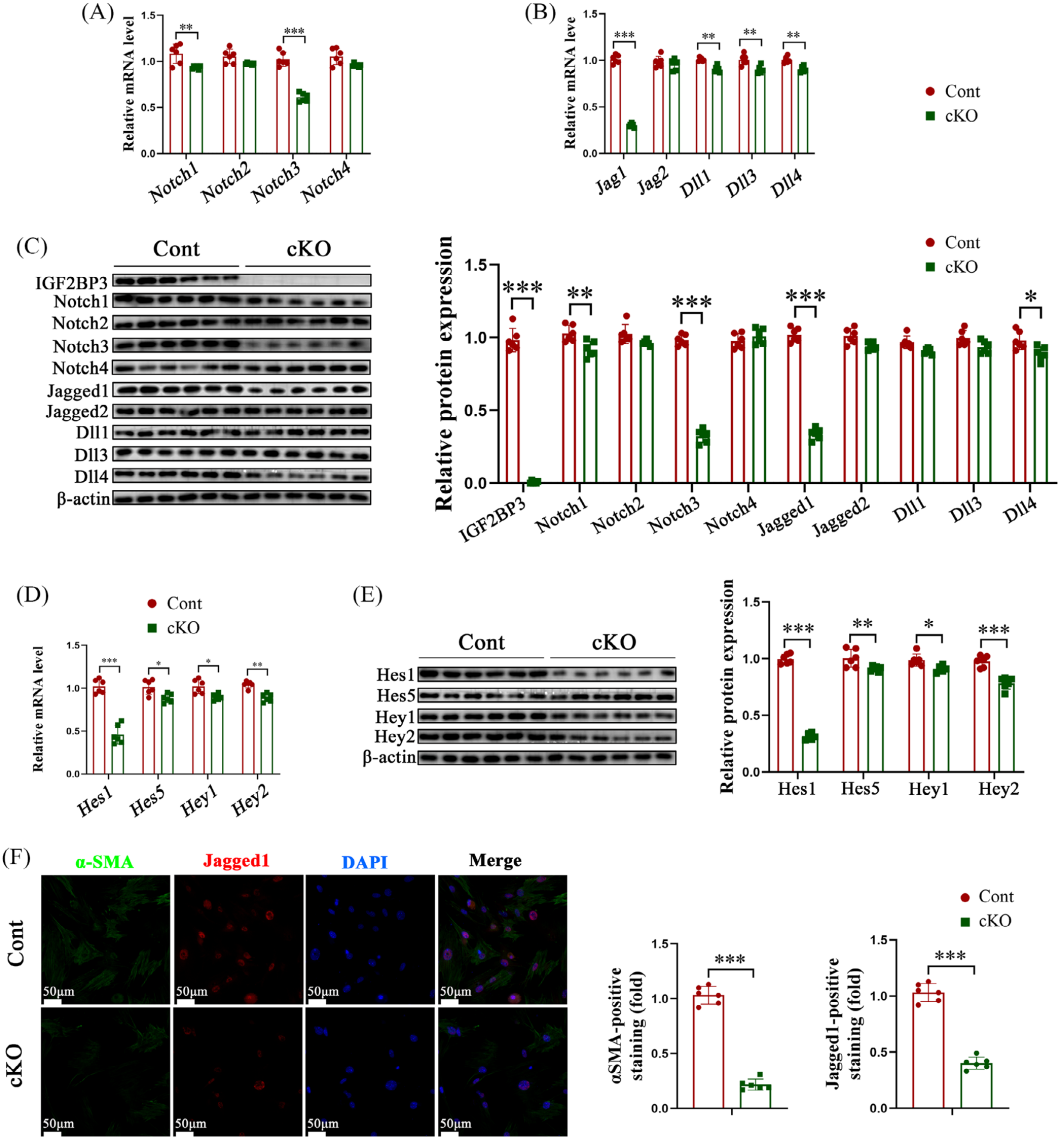
IGF2BP3 knockout suppresses the Notch/Jag1/Hes1 signalling. Primary HSCs were isolated from control and IGF2BP3 cKO mice. (A,B) mRNA levels of Notch receptors and ligands. (C) Protein levels of Notch receptors and ligands. (D,E) Levels of Hes1, Hes5, Hey1 and Hey2. (F) Immunofluorescence staining for α‐SMA and Jagged1. Data are presented as mean ± SD from six experiments. *p < .05, **p < .01, ***p < .001; SD, standard deviation.

Aberrant Notch signalling activation is often linked to its receptors and ligands. Silencing IGF2BP3 significantly reduced the expression of Jag1 and Notch3 (Figure 7A–C). Additionally, the protein levels of downstream molecules in the Notch‐Jag1 signalling pathway, such as Hes1, Hes5, Hey1, and Hey2, were significantly decreased upon IGF2BP3 knockdown (Figure 7D,E). Immunofluorescence analysis confirmed that Jag1 and α‐SMA levels were markedly attenuated after IGF2BP3 knockdown (Figure 7F). Similar reductions in Jag1, Notch3 and Hes1 levels were observed in LX2 cells post‐IGF2BP3 inhibition (Figure S4). RIP‐qPCR experiments demonstrated the direct binding between IGF2BP3 and Jag1 (Figure S4B). These findings implicate Notch‐Jag1 signalling in the regulation of HSC activation by IGF2BP3 deficiency‐mediated m^6^A modifications.

### 3.5 Jag1 overexpression suppresses IGF2BP3 KO‐mediated ferroptosis of hepatic stellate cells

**FIGURE 8:**
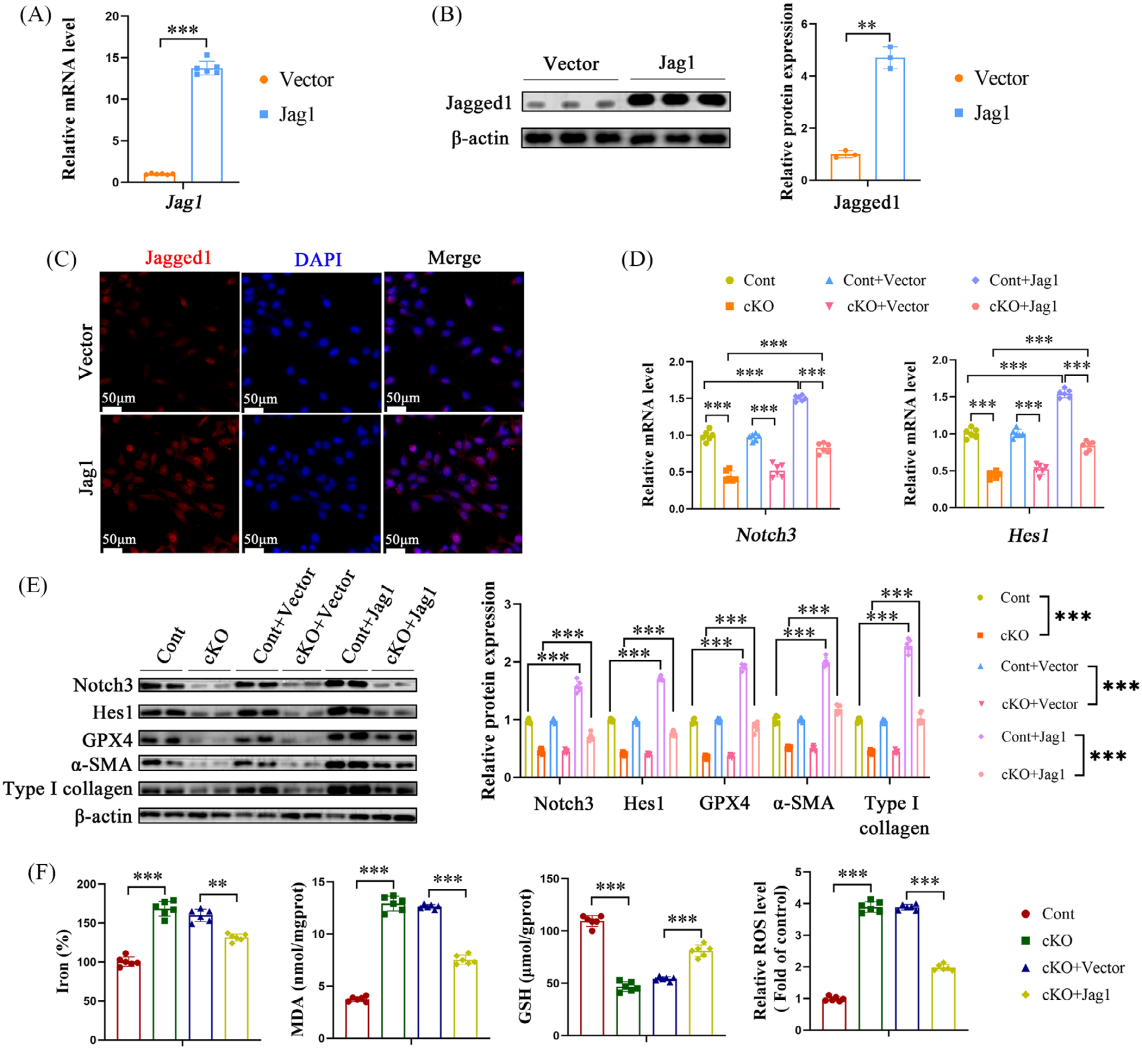
Overexpression of Jag1 inhibits the effects of IGF2BP3 knockdown on HSC inactivation and FPT. Primary HSCs isolated from control and IGF2BP3 cKO mice were transduced with Jag1 overexpression plasmids. (A) Jag1 mRNA levels. (B) Jagged1 protein levels. (C) Immunofluorescence staining of Jagged1. (D) mRNA levels of Notch3 and Hes1. (E) Protein levels of Notch3, Hes1, GPX4, α‐SMA and Type I collagen. (F) Levels of iron, MDA, GSH and ROS. Data are presented as mean ± SD from six experiments. **p < .01, ***p < .001. HSC, Hepatic stellate cell; MDA, malondialdehyde; GSH, glutathione; ROS, reactive oxygen species; FPT, ferroptosis; SD, standard deviation.

To further verify the role of Jag1 in counteracting the effects of IGF2BP3 cKO‐induced m^6^A modifications on HSC activation, Jag1 was overexpressed, leading to elevated Jag1 levels in cells (Figure 8A–C). This overexpression increased Notch3 and Hes1 levels in primary HSCs (Figure 8D,E) and reversed the IGF2BP3 cKO‐induced suppression of GPX4 (Figure 8E). Additionally, Jag1 overexpression mitigated the IGF2BP3 cKO‐induced rise in FPT indicators, including iron, MDA, ROS, and depleted GSH (Figure 8F). Similar inhibitory effects on Notch signalling and HSC FPT were observed in LX2 cells following IGF2BP3 knockdown and subsequent Jag1 overexpression (Figure S5). These results indicate that the loss of IGF2BP3 inhibits HSC activation and promotes the FPT of HSCs by downregulating Jag1.

### 3.6 IGF2BP3 inhibition facilitates ferroptosis of hepatic stellate cells via the Hes1/GPX4 axis

**FIGURE 9:**
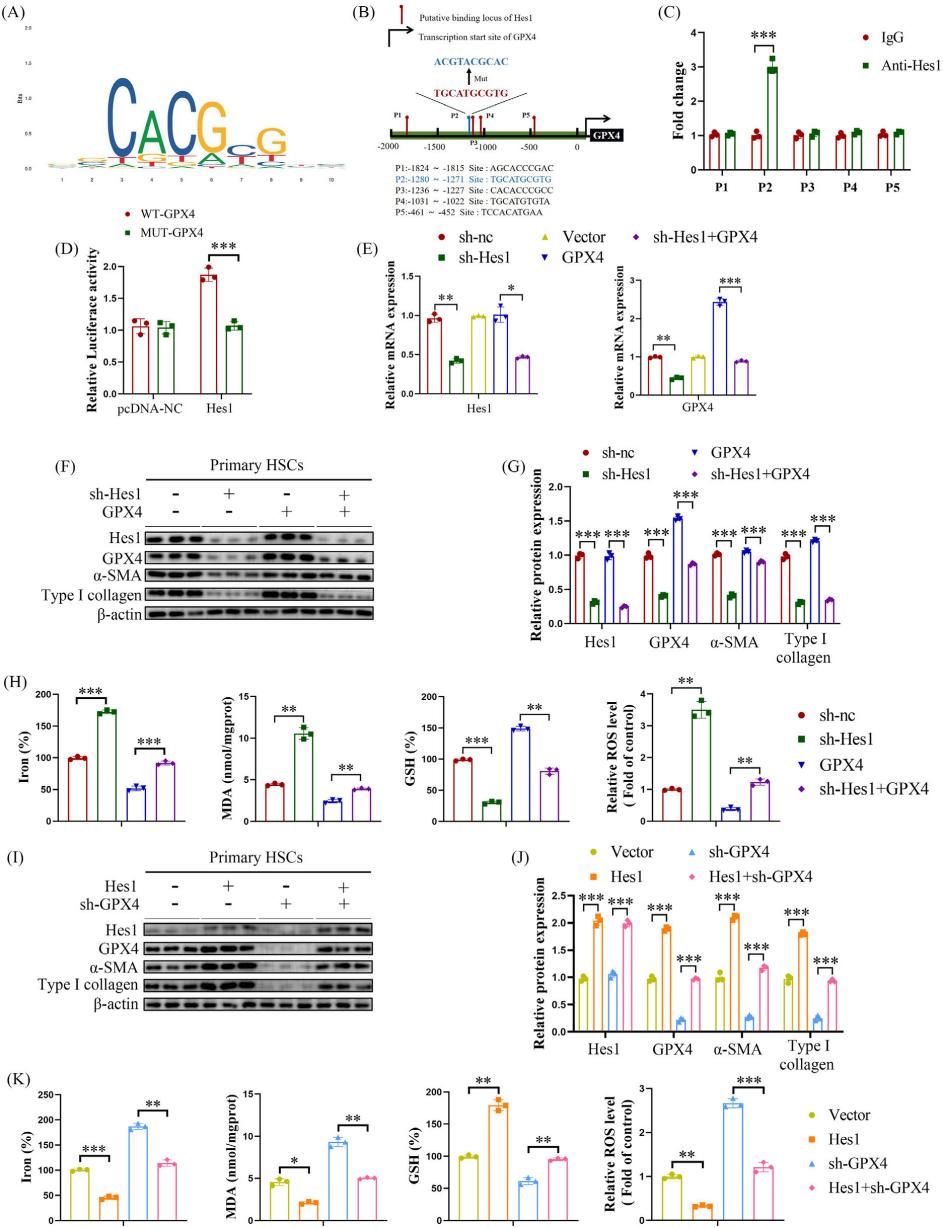
IGF2BP3 inhibition facilitates HSC FPT via the Hes1/GPX4 axis. Knockdown of Hes1 and GPX4 or overexpression of GPX4 and Hes1 were performed in primary HSCs isolated from control mice. (A) Hes1‐binding sites in the GPX4 promoter predicted by JASPAR (http://jaspar.genereg.net/). (B) Schematic representation of the potential Hes1 binding sites in the GPX4 promoter. (C) ChIP analysis of Hes1 occupancy at the GPX4 promoter. (D) Luciferase reporter assays. (E) mRNA levels of Hes1 and GPX4. (F,G,I, J) Protein levels of Hes1, GPX4, α‐SMA and Type I collagen. (H,K) Levels of iron, MDA, GSH, and ROS. Data are presented as mean ± SD from three experiments. *p < .05, **p < .01, ***p < .001. HSC, Hepatic stellate cell; MDA, malondialdehyde; GSH, glutathione; ROS, reactive oxygen species; FPT, ferroptosis; SD, standard deviation; ChIP, chromatin immunoprecipitation.

Downstream molecules of the Notch pathway, such as Hes1, Hes5, Hey1 and Hey2, function as transcription factors. Amongst these, only Hes1 inhibition reduced GPX4 levels (Figure S6), prompting further exploration of the Hes1–GPX4 interaction. JASPAR analysis identified five putative motifs (P1–P5) for Hes1 binding to the GPX4 promoter (Figure 9A,B). Chromatin immunoprecipitation‐quantitative PCR indicated that the P2 motif is the likely Hes1 binding site (Figure 9C). Luciferase reporter assays confirmed that Hes1 upregulates GPX4‐WT, but not GPX4‐MUT (with a mutated P2 site), verifying Hes1 interaction with GPX4 at the P2 site (Figure 9D). Further experiments demonstrated Hes1's vital role in HSC FPT. Similar to Jag1, Hes1 loss inhibited HSC activation and promoted HSC FPT, reversible by GPX4 overexpression (Figure 9E–H). Conversely, Hes1 overexpression enhanced HSC activation and suppressed HSC FPT, which was reversed by GPX4 knockout (Figure 9I–K). These results suggest that IGF2BP3 inhibition facilitates HSC FPT, at least in part via the Hes1/GPX4 axis.

### 3.7 IGF2BP3 levels correlate with cirrhosis parameters in humans

Finally, the relevance of these findings to human pathology was investigated. qPCR analysis of liver specimens from patients with cirrhosis and healthy donors revealed significantly higher IGF2BP3 levels in cirrhotic patients (Figure S7A). Elevated IGF2BP3 levels were associated with increased liver injury and fibrosis, as indicated by Col1A1 expression (Figure S7B–D), suggesting that IGF2BP3 elevation may contribute to cirrhosis pathogenesis in humans.

## 4 DISCUSSION

The liver fibrosis development is primarily driven by HSC activation. Following injury, HSCs differentiate into activated myofibroblasts, which are responsible for the secretion and accumulation of fibrillar collagen. Therefore, inhibiting HSC activation represents a promising therapeutic strategy for treating liver fibrosis.^18^ The m^6^A modification of RNA plays a pivotal role in various physiological and pathophysiological processes in mammals.^19^ Our findings demonstrate that the loss of IGF2BP3 exerts an inhibitory effect on liver fibrosis, consistent with the results reported by Wang et al.^20^ Whilst Wang et al. showed that IGF2BP3 loss leads to decreased HSC proliferation, the specific mechanisms were not fully explored. Our study suggests that knocking out IGF2BP3 promotes FPT of HSCs and inhibits their activation and proliferation, thereby preventing liver fibrosis progression. This highlights IGF2BP3‐induced m^6^A modifications as promising targets for liver fibrosis amelioration.

Evidence increasingly supports the use of FPT to treat and prevent liver fibrosis.^21^, ^22^ Wang et al. found that FPT in hepatocytes hinders liver injury recovery.^23^ Thus, inducing FPT in HSCs, rather than hepatocytes, presents a novel approach to treating liver fibrosis. IGF2BP3 inhibition was found to promote FPT in HSCs, primarily by damaging mitochondrial cristae. In vitro analysis of FPT‐related factors such as GPX4, ACSL4, and SCL7A11 revealed that GPX4, an antioxidant enzyme that maintains ROS balance and negatively regulates FPT, plays a significant role in IGF2BP3 deficiency‐induced FPT in HSCs. The effects of IGF2BP3 deficiency on FPT and GPX4 levels were notably suppressed by Fer‐1, suggesting that IGF2BP3 deficiency promotes FPT in HSCs at least partially through GPX4. The m^6^A modification correlates with various human diseases and influences cell fate by affecting apoptosis, necroptosis, senescence and epithelial–mesenchymal transition.^24^, ^25^ IGF2BP3, a key m^6^A reader, significantly impacts gene regulation in human diseases. Yan et al. demonstrated that suppressing IGF2BP3 inhibits HCC cell migration and invasion,^26^ whilst Deng et al. showed that IGF2BP3 loss induces FPT in glioblastoma by directly regulating GPX4 expression.^27^ Lu et al. established that IGF2BP3 can regulate HCC FPT.^7^ However, its effect on HSC FPT remained unclear. This study established that IGF2BP3 downregulation and m^6^A are associated with HSC FPT. Although further research is necessary to fully elucidate m^6^A's influence on FPT, these results highlight a novel role for m^6^A in HSC FPT. Omics assays revealed that Jag1, a Notch signalling ligand, mediates the impact of IGF2BP3‐induced m^6^A modifications on HSC activation. These results align with reports underscoring the critical role of Jag1 and Notch signalling in liver fibrosis.^28^, ^29^ Upregulated Jag1 during HSC activation has been observed in both animal and human models, consistent with our findings.^29^, ^30^ Interestingly, Jag1 promotes HSC activation by signalling through Notch1 and Notch3, rather than Notch2 or Notch4, reflecting the results of Kong et al.^28^ Yang et al. demonstrated that HSC‐specific Notch1 deletion in mice confers resistance to bile duct ligation‐induced liver fibrosis.^31^ This study linked IGF2BP3 depletion to Notch‐Jag1 signalling in HSC activation and liver fibrosis development. Antibodies targeting Jag1 and Notch1 have shown remarkable potential in treating N‐Ras‐induced primary liver cancer in mice.^32^ Utilising similar antibodies targeting IGF2BP3, Jag1, Notch1/3, and Hes1 as a therapeutic strategy in pre‐existing liver fibrosis models would enhance the translational value of these discoveries.

The relationship between IGF2BP3‐mediated Notch signalling and FPT in HSCs was further explored. qRT‐PCR and Western blotting identified probable downstream signalling pathways associated with Jag1. IGF2BP3 knockdown significantly decreased the expression of Notch1/3 and Hes1. Notch1/3's essential role in liver pathophysiology is well‐documented. Chen et al. reported activated Notch signalling in the CCl_4_ model of liver fibrosis, marked by upregulated Notch3 and Hes1, consistent with our findings.^33^ Hes1, a member of the basic helix–loop–helix family of transcription factors, functions by binding to N‐boxes (CACNAG) and is crucial for cell proliferation, differentiation and liver fibrogenesis.^34^, ^35^, ^36^ Chromatin immunoprecipitation and luciferase assays in this study suggested that Hes1 may directly bind to the GPX4 promoter, implying that Hes1 induces HSC FPT by binding to the GPX4 promoter, a key negative regulator of FPT.

Our study has several strengths and weaknesses. Using three methods to demonstrate that HSC IGF2BP3 knockdown attenuates liver fibrosis—short hairpin RNAs (shIGF2BP3), the BETi JQ1, and *IGF2BP3^−/−^* mice—enhances the reliability of our results. Despite significant discoveries, the study has limitations and unanswered questions. First, m^6^A, the most common internal RNA modification, is crucial in regulating the RNA metabolism of both protein‐coding and non‐coding RNAs.^37^ Hence, targeting m^6^A might inhibit HSC activation and liver fibrosis by regulating other protein‐coding or non‐coding targets. Second, other aspects of IGF2BP3's profibrotic effect in HSCs, such as inflammation signalling and microRNA release, require further investigation. Third, the CCl_4_ mouse model showed increased liver and body weight following IGF2BP3 cKO, suggesting that IGF2BP3 deletion may also promote liver regeneration, a mechanism that needs further study. Lastly, incorporating human primary HSCs in experiments could provide better insights into the applicability of this mechanism in humans.

In summary, our data demonstrate that IGF2BP3 deficiency promotes HSC FPT and inactivation. Furthermore, targeting the IGF2BP3/Notch/Jag1 signalling axis presents a promising therapeutic approach for treating liver fibrosis. Developing specific antibodies against IGF2BP3 for future studies on liver fibrosis could significantly enhance the translational potential of our current findings.

## AUTHOR CONTRIBUTIONS

**Jianjian Zheng**: Designed the study. **Xinmiao Li and Weizhi Zhang**: Performed the majority of the experiments. **Feng Jiang; Yining Wang and Lifan Lin**: Analysed data and wrote the manuscript. **Yifei Li; Lingling Wu and Han Zeng**: Conducted revisions and added experiments. All authors contributed to the paper and approved the submitted version.

## CONFLICT OF INTEREST STATEMENT

The authors declare no known financial conflicts of interest or personal relationships that could have influenced the findings reported in this paper.

## ETHICS STATEMENT

All procedures involving human samples were conducted in accordance with the principles outlined in the Declaration of Helsinki and received approval from the FAH Ethics Committee (KY2021‐177), with the approval date being 18 November, 2021. The Laboratory Animal Ethics Committee of Wenzhou Medical University approved all animal procedures conducted in this study (No. wydw2023‐0498).

## Supporting information

Supporting information

Supporting information

Supporting information

Supporting information

Supporting information

Supporting information

Supporting information

Supporting information

## Data Availability

The data used and/or analysed during this study are available from the corresponding author upon reasonable request.
