# Supplementary material for: The IGF2BP3/Notch/Jag1 pathway: A key regulator of hepatic stellate cell ferroptosis in liver fibrosis
Source: Clin Transl Med. 2024 Aug 7;14(8):e1793. doi: 10.1002/ctm2.1793 (PMC11306284; doi:10.1002/ctm2.1793)
Supplement: Supplementary file 1 — Supporting information [file CTM2-14-e1793-s007.doc]

**Supporting Information**

**Author-Supplied**

Xinmiao Li^a^, Yifei Li^a^, Weizhi Zhang^a^, Feng Jiang^a^, Lifan Lin^a^, Yining Wang^b^, Lingling Wu^c^, Han Zeng^a^, Jianjian Zheng^a,*^

^a^Zhejiang Key Laboratory of Intelligent Cancer Biomarker Discovery and Translation, The First Affiliated Hospital of Wenzhou Medical University, Wenzhou, China

^b^School of Mental Health, Wenzhou Medical University, Wenzhou, China

^c^Wenzhou Medical University Renji College, Wenzhou, China

**Funding Information**

The project was supported by the National Natural Science Foundation of China (No. 81873576) and Wenzhou Municipal Science and technology Bureau (No.Y20220023).


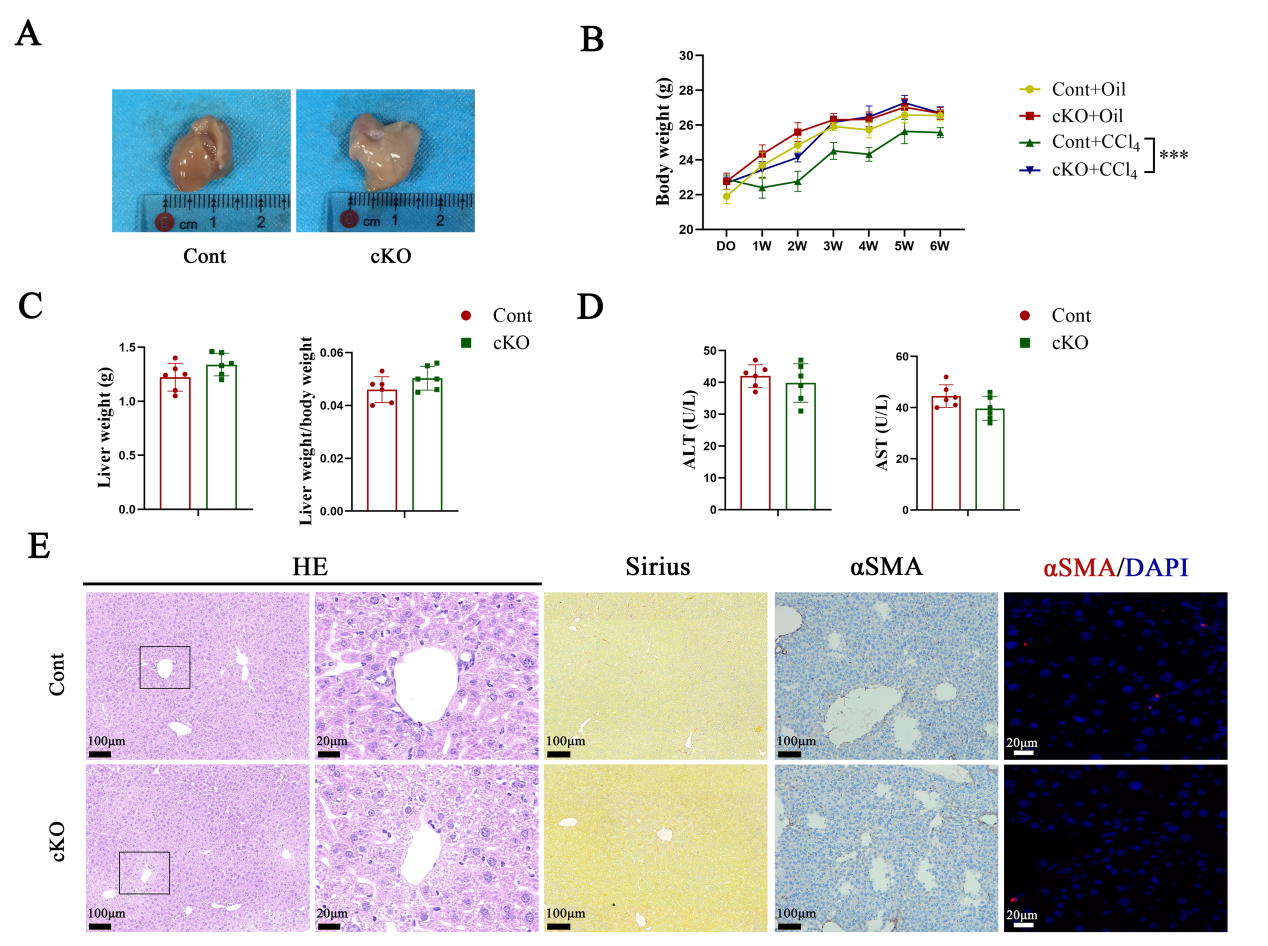
Figure S1 Related to Figure 1. HSC-specific knockout of IGF2BP3 does not affect mouse development.

(A) Representative gross appearance of livers. (B, C) The body weight, liver weight, and liver weight to body weight ratio. (D) Levels of ALT and AST. (E) HE staining, Sirius Red, α-SMA immunohistochemical staining and immunofluorescence staining. Each value is the mean ± SD of six experiments. ****P*< 0.001.


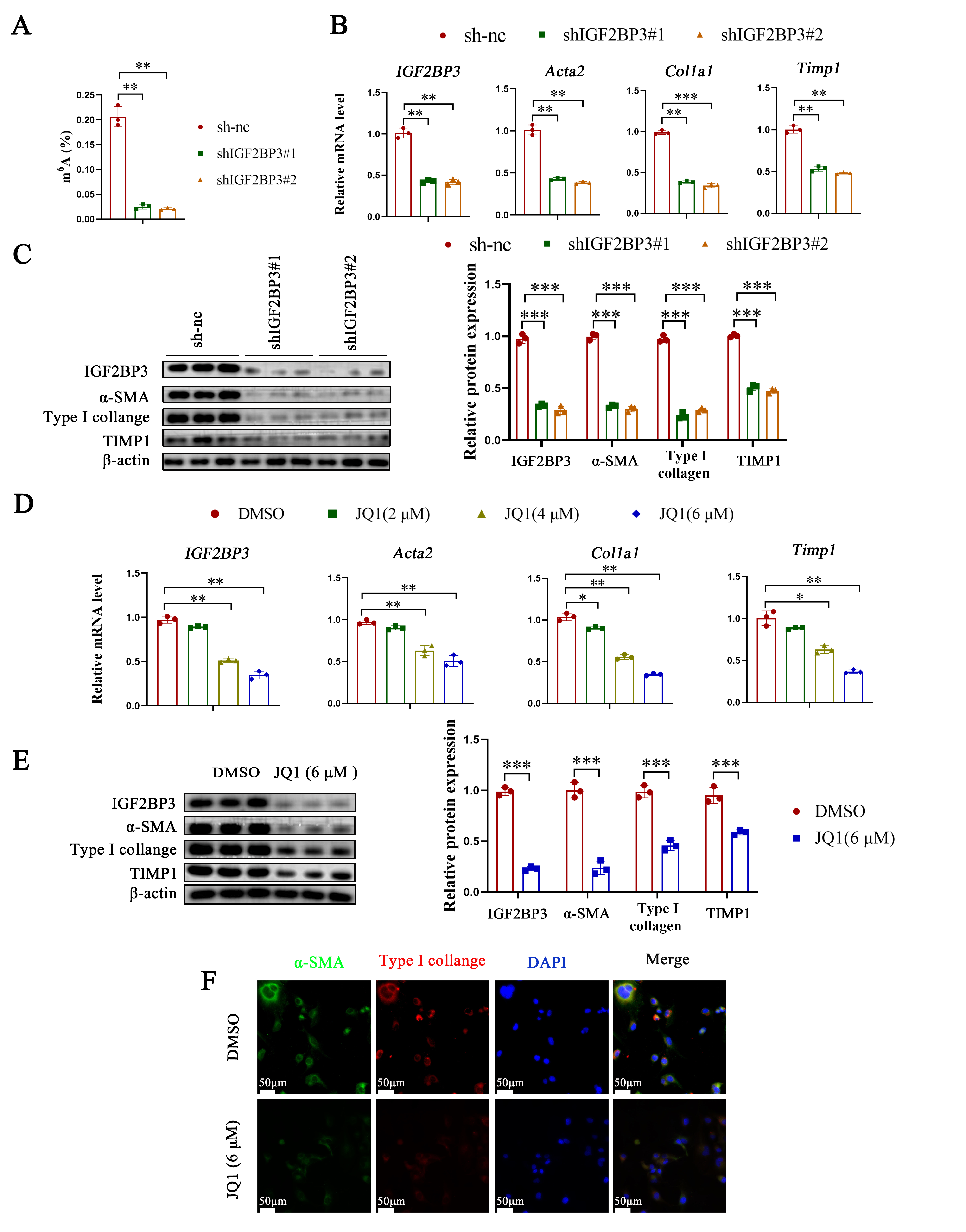


Figure S2 Related to Figure 4. Inhibition of IGF2BP3 blocks the activation and expression of pro-fibrosis markers in LX2 cells

LX2 cells were stably transfected with IGF2BP3 shRNA or treated with JQ1 for 24 h.

(A) m6A levels. (B and C) Levels of IGF2BP3 and pro-fibrosis markers in HSCs with IGF2BP3 shRNA. (D and E) Levels of IGF2BP3 and pro-fibrosis markers in HSCs with JQ1 treatment. (F) Immunofluorescence staining of α-SMA and Type I collagen. Each value is the mean ± SD of three experiments. **P*< 0.05, ***P*< 0.01, ****P*< 0.001.


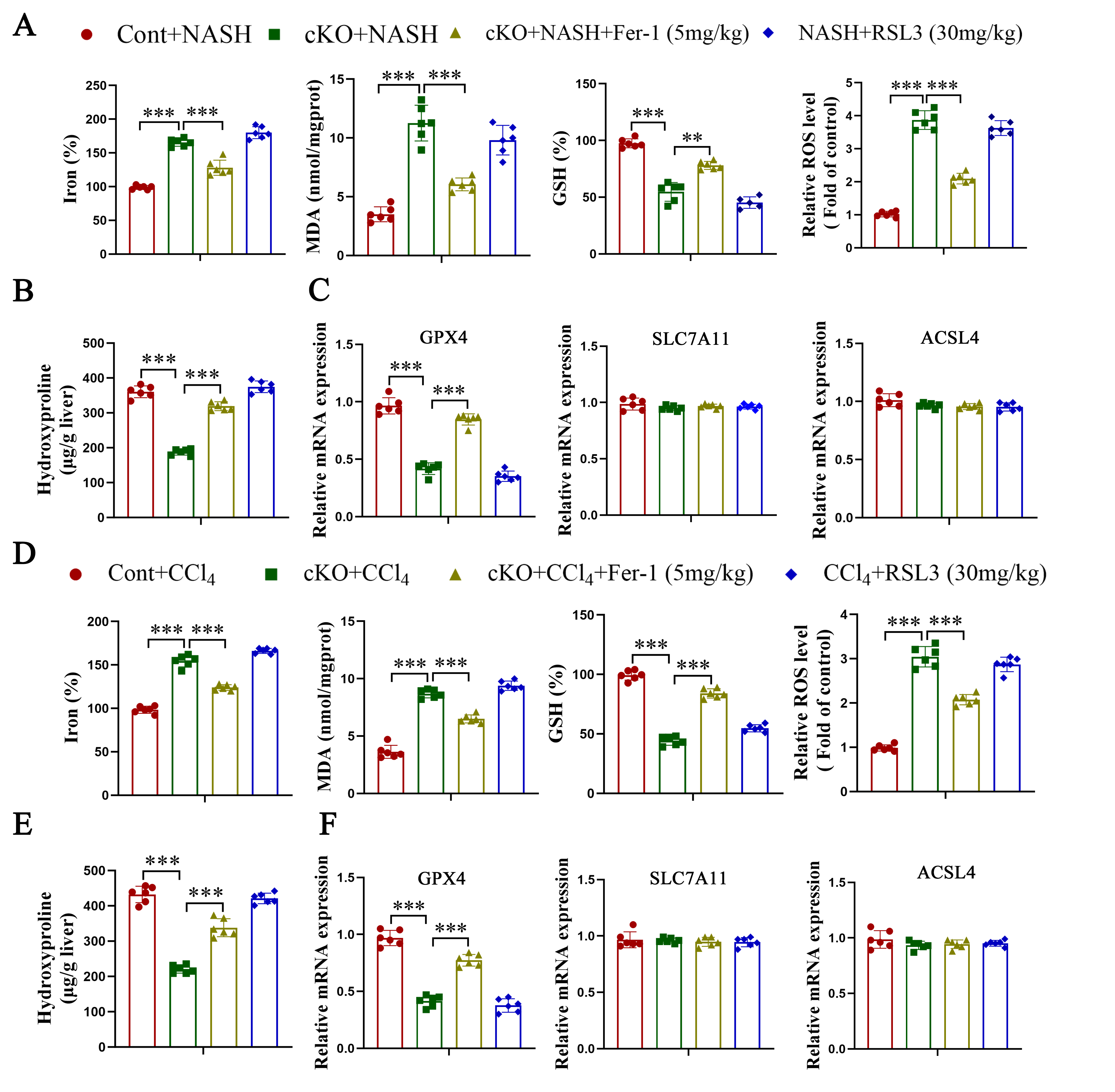


Figure S3 Related to Figure 5. HSC-specific knockout of IGF2BP3 enhances HSC FPT via GPX4 *in vivo*

(A) Iron, MDA and GSH, ROS levels. (B) Hyp level. (C) mRNA levels of FPT-related gene. (D) Iron, MDA and GSH, ROS levels. (E) Hyp level. (F) mRNA levels of FPT-related gene. Each value is the mean ± SD of six experiments. ***P*< 0.01, ****P*< 0.001.


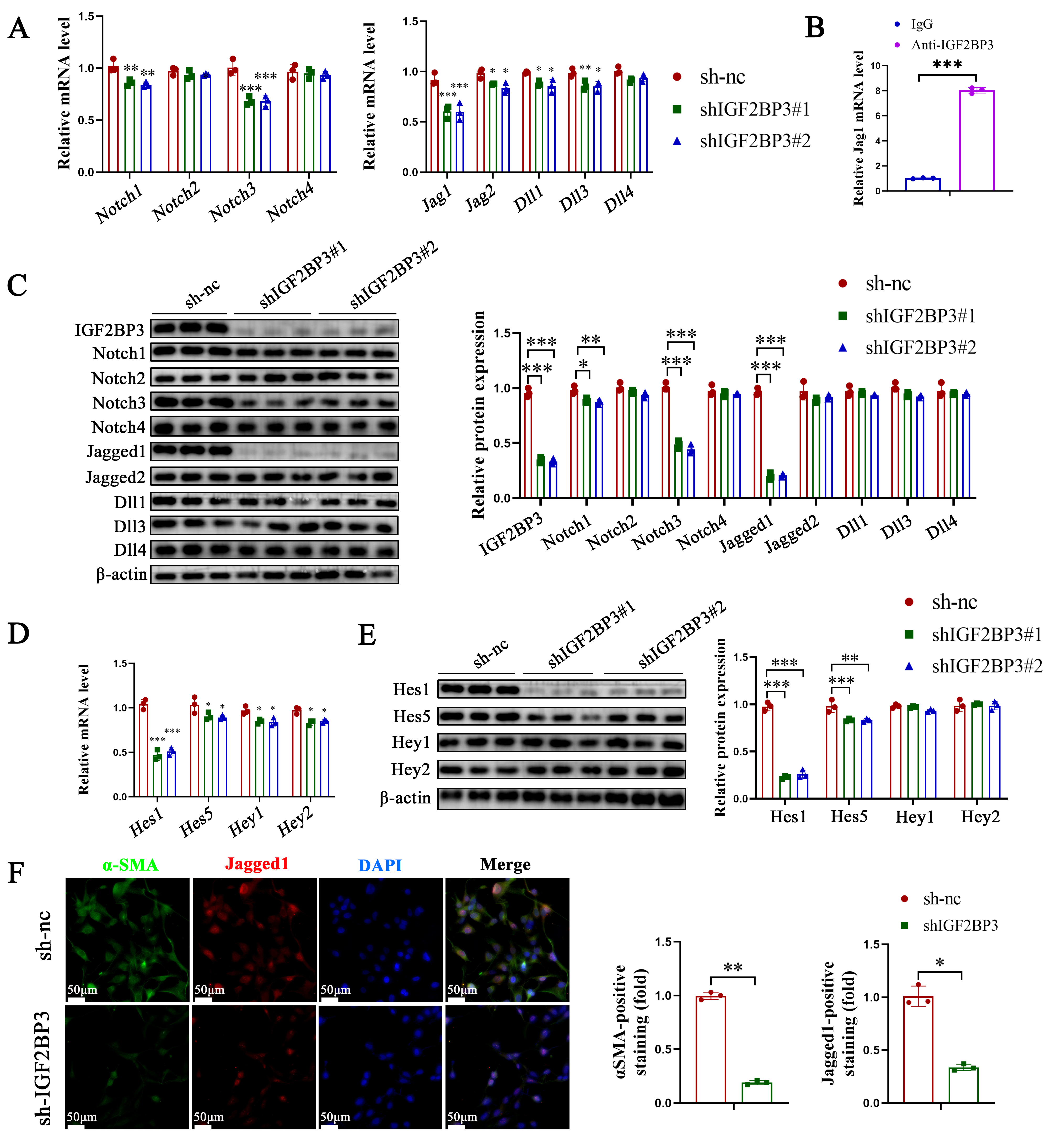


Figure S4 Related to Figure 7. IGF2BP3 knockdown suppressed the Jag1/Notch3/Hes1 signalling pathway in LX2 cells.

(A ) mRNA and protein levels of Notch receptor and ligand. (B) RIP‐qPCR detecting binding of IGF2BP3 to the Jag1. (C) Protein levels of Notch receptor and ligand. (D and E) Levels of Hes1, Hes5, Hey1, and Hey2. (F) Immunofluorescence staining for α-SMA and Jagged1. Each value is the mean ± SD of six experiments. **P*< 0.05, ***P*< 0.01, ****P*< 0.001.


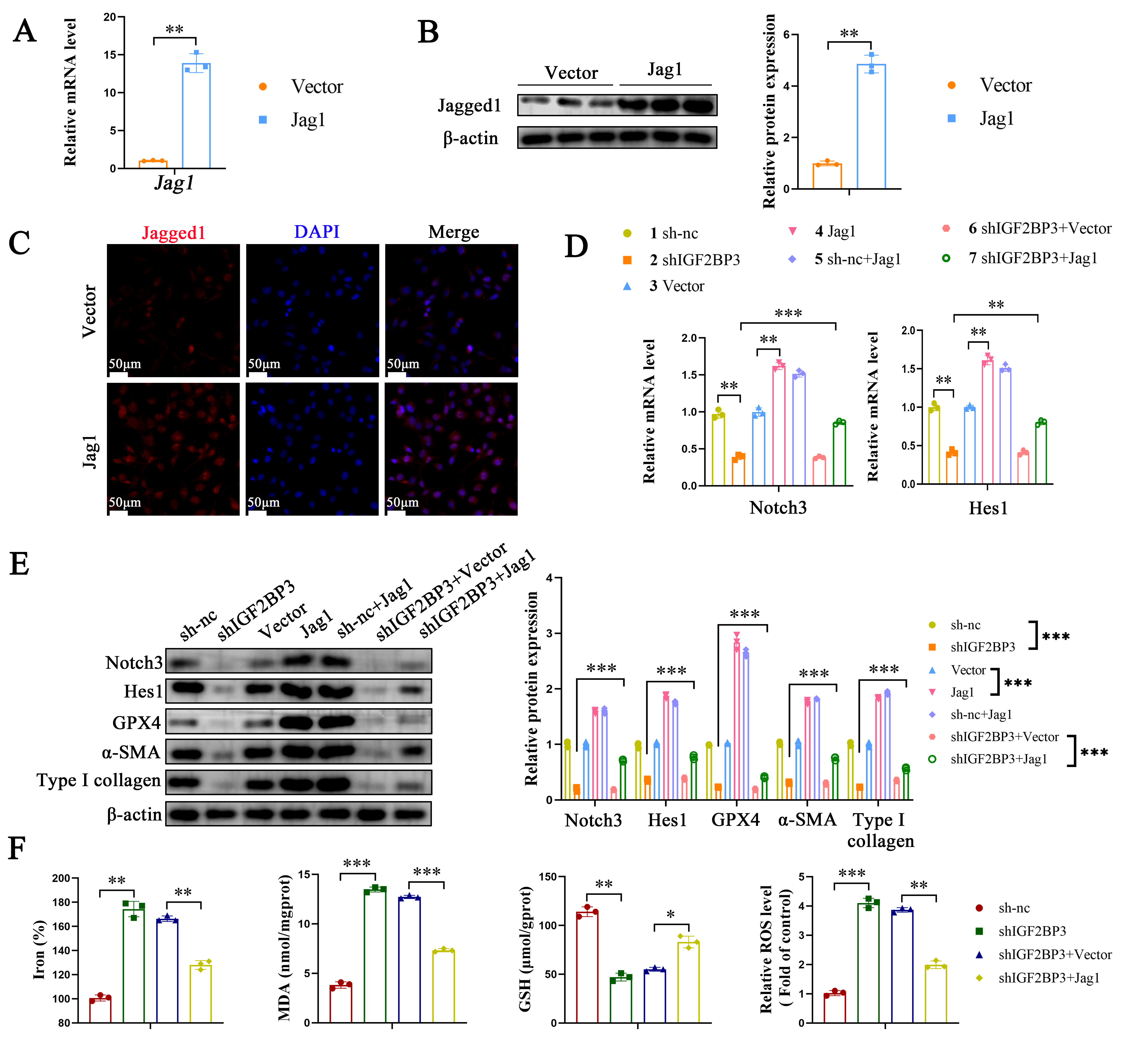


Figure S5 Related to Figure 8. Overexpression of Jag1 inhibits the effects of IGF2BP3 knockdown LX2 cells activation.

LX2 cells were transduced with pLVX-Puro plasmids of Jag1 overexpression.

(A) Jag1 mRNA. (B) Jagged1 protein. (C) Immunofluorescence staining of Jagged1. (D) mRNA levels of Notch3 and Hes1. (E) Protein levels of Notch3, Hes1, GPX4, α-SMA and Type I collagen. (F) Iron, MDA and GSH, ROS levels. Each value is the mean ± SD of six experiments. **P*< 0.05, ***P*< 0.01, ****P*< 0.001.


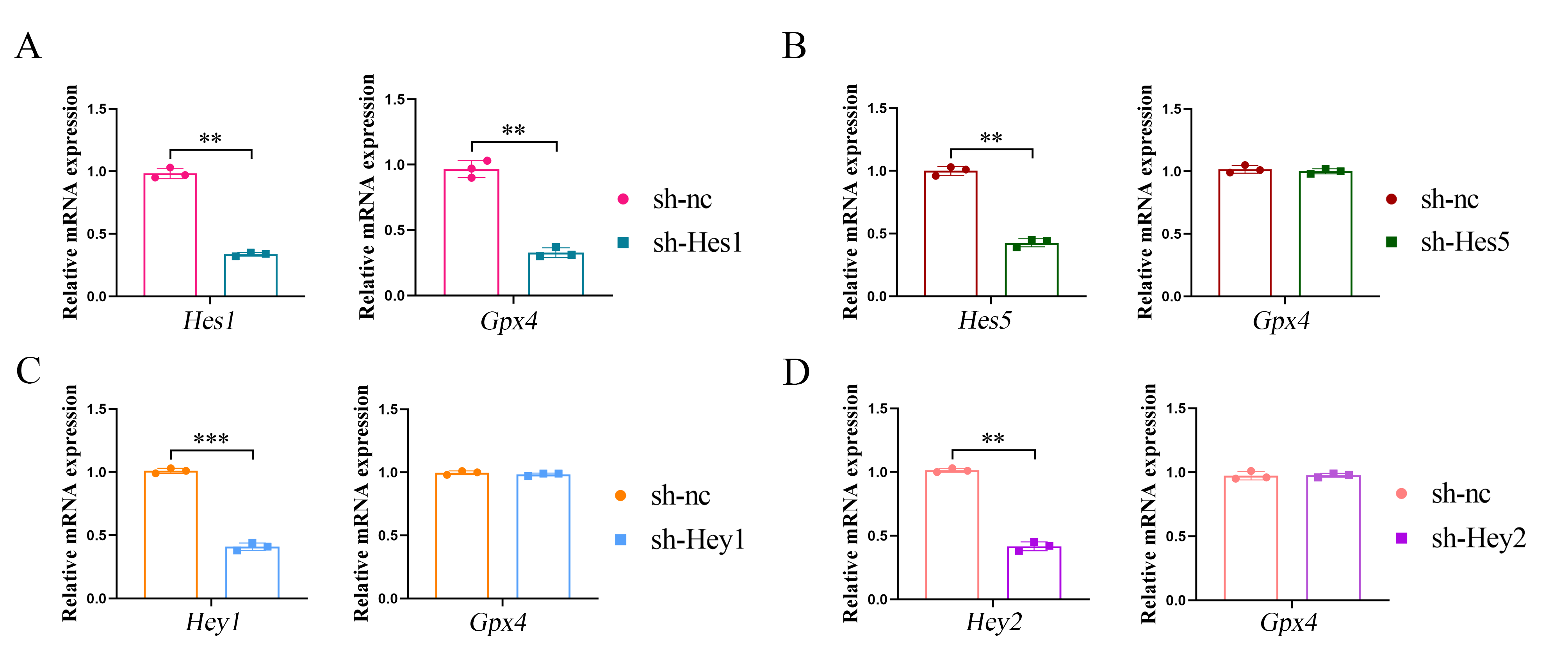


Figure S6 Related to Figure 9. Hes1 expression is correlated with GPX4 expression in primary HSCs.

1. mRNA levels of Hes1 and GPX4. (B) mRNA levels of Hes5 and GPX4. (C) mRNA levels of Hey1 and GPX4. (D) mRNA levels of Hey2 and GPX4. Each value is the mean ± SD of three experiments. ***P*< 0.01, ****P*< 0.001.


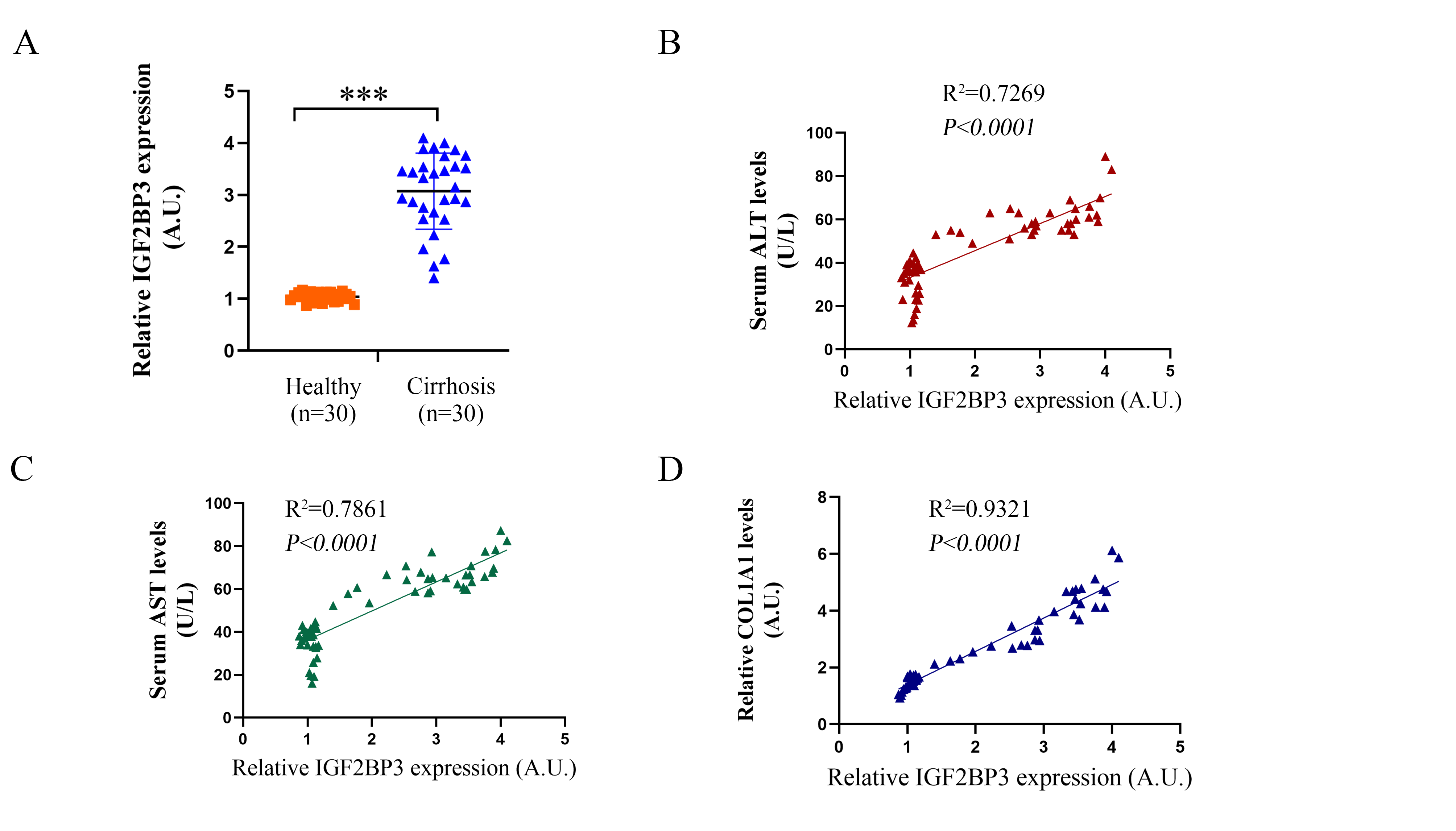


Figure S7 Related to Figure 9. IGF2BP3 levels correlate with cirrhosis parameters in humans.

(A) IGF2BP3 expression levels in the livers of healthy individuals and cirrhosis patients were examined by qPCR. (B-D) Correlations between IGF2BP3 and serum ALT/AST/COL1A1 levels were evaluated by linear regression (n=60). ****P*< 0.001.

| Table S1 | | |
| --- | --- | --- |
| **1.** The primers used for qRT-PCR (mouse) | | |
| Primer | Sense | Antisense |
| IGF2BP3 | 5'-ACGAAATATCCCGCCTCATTTAC-3' | 5'-GCAGTTTCCGAGTCAGTGTTCA-3' |
| α-SMA | 5'-GCCATCTTTCATTGGGATGGA-3' | 5'-CCCCTGACAGGACGTTGTTA-3' |
| Col1A1 | 5'-GCTCCTCTTAGGGGCCACT-3' | 5'-CCACGTCTCACCATTGGGG-3' |
| TIMP1 | 5'-GCTAAAAGGATTCAAGGC-3' | 5'-GCACAAGCCTAGATTCCG-3' |
| Notch1 | 5'-GATGGCCTCAATGGGTACAAG-3' | 5'-TCGTTGTTGTTGATGTCACAGT-3' |
| Notch2 | 5'-GACTGCCAATACTCCACCTCT-3' | 5'-CCATTTTCGCAGGGATGAGAT-3' |
| Notch3 | 5'-AGTGCCGATCTGGTACAACTT-3' | 5'-CACTACGGGGTTCTCACACA-3' |
| Notch4 | 5'-GAACGCGACATCAACGAGTG-3' | 5'-GGAACCCAAGGTGTTATGGCA-3' |
| Jag1 | 5'-AATCGCATCGTACTGCCTTTC-3' | 5'-GTGTCATTACTGGAATCCCAGG-3' |
| Jag2 | 5'-GCGACCAGTACGGCAACAA-3' | 5'-CCGTGGAGCAAATTACATCCTT-3' |
| Dll1 | 5'-CCCATCCGATTCCCCTTCG-3' | 5'-GGTTTTCTGTTGCGAGGTCATC-3' |
| Dll3 | 5'-GCTGGTGTCTTCGAGCTACAA-3' | 5'-TGCTCCGTATAGACCGGGAC-3' |
| Dll4 | 5'-CAGTTGCCCTTCAATTTCACCT-3' | 5'-AGCCTTGGATGATGATTTGGC-3' |
| Hes1 | 5'-TCAACACGACACCGGACAAAC-3' | 5'-ATGCCGGGAGCTATCTTTCTT-3' |
| Hes5 | 5'-AGTCCCAAGGAGAAAAACCGA-3' | 5'-GCTGTGTTTCAGGTAGCTGAC-3' |
| Hey1 | 5'-CCGACGAGACCGAATCAATAAC-3' | 5'-TCAGGTGATCCACAGTCATCTG-3' |
| Hey2 | 5'-CGCCCTTGTGAGGAAACGA-3' | 5'-CCCAGGGTAATTGTTCTCGCT-3' |
| GPX4 | 5'-ACGAGTTCCTGGGCTTGTGT-3' | 5'-TGCGAATTCGTGCATGGA-3' |
| SCL7A11 | 5'-GGCTCCATGAACGGTGGTGTG-3' | 5'-GCTGGTAGAGGAGTGTGCTTGC-3' |
| ACSL4 | 5'-ACTGGCGATATTGGAGAAT-3' | 5'-CACATAGGACTGGTCACTT-3' |
| β-actin | 5'-GGCTGTATTCCCCTCCATCG-3' | 5'-CCAGTTGGTAACAATGCCATGT-3' |

| **2.** The primers used for qRT-PCR (human) | | |
| --- | --- | --- |
| Primer | Sense | Antisense |
| IGF2BP3 | 5'-GCACTTCCCTTTGTTGTAGTC-3' | 5'-AGCACTTCCCTTAGGTTACTC-3' |
| α-SMA | 5'-GTGTTGCCCCTGAAGAGCAT-3' | 5'-GCTGGGACATTGAAAGTCTCA-3' |
| Col1A1 | 5'-CCCGGGTTTCAGAGACAACTTC-3' | 5'-TCCACATGCTTTATTCCAGCAATC-3' |
| TIMP1 | 5'-CTTCTGCAATTCCGACCTCGT-3' | 5'-ACGCTGGTATAAGGTGGTCTG-3' |
| Notch1 | 5'-GAGGCGTGGCAGACTATGC-3' | 5'-CTTGTACTCCGTCAGCGTGA-3' |
| Notch2 | 5'-CAACCGCAATGGAGGCTATG-3' | 5'-GCGAAGGCACAATCATCAATGTT-3' |
| Notch3 | 5'-TGGCGACCTCACTTACGACT-3' | 5'-CACTGGCAGTTATAGGTGTTGAC-3' |
| Notch4 | 5'-TGTGAACGTGATGTCAACGAG-3' | 5'-ACAGTCTGGGCCTATGAAACC-3' |
| Jag1 | 5'-GCCGAGGTCCTATACGTTGC-3' | 5'-CCGAGTGAGAAGCCTTTTCAA-3' |
| Jag2 | 5'-TGGGCGGCAACTCCTTCTA-3' | 5'-GCCTCCACGATGAGGGTAAA-3' |
| Dll1 | 5'-GATTCTCCTGATGACCTCGCA-3' | 5'-TCCGTAGTAGTGTTCGTCACA-3' |
| Dll3 | 5'-CACTCCCGGATGCACTCAAC-3' | 5'-GATTCCAATCTACGGACGAGC-3' |
| Dll4 | 5'-GTCTCCACGCCGGTATTGG-3' | 5'-CAGGTGAAATTGAAGGGCAGT-3' |
| Hes1 | 5'-ACGTGCGAGGGCGTTAATAC-3' | 5'-GGGGTAGGTCATGGCATTGA-3' |
| Hes5 | 5'-GGAGAAAAACCGACTGCGGAA-3' | 5'-ATCTCCAGGATGTCGGCCT-3' |
| Hey1 | 5'-GTTCGGCTCTAGGTTCCATGT-3' | 5'-CGTCGGCGCTTCTCAATTATTC-3' |
| Hey2 | 5'-AAGGCGTCGGGATCGGATAA-3' | 5'-AGAGCGTGTGCGTCAAAGTAG-3' |
| GPX4 | 5'-TTCCCGAACTGGTTACACGG-3' | 5'-GTTTTCCGCCAAGGACATCG-3' |
| β-actin | 5'-GGCACCCAGCACAATGAAG-3' | 5'-CCGATCCACACGGAGTACTTG-3' |

| Table S2 | | |
| --- | --- | --- |
| **1.** Antibodies for Western blot | | |
| Antibodies | Source | Identifier |
| IGF2BP3 | Proteintech, Chicago, USA | 14642-1-AP |
| α-SMA | Abcam, Cambridge, UK | ab124964 |
| Type I clollagen (human) | Abcam, Cambridge, UK | ab138492 |
| Type I clollagen (mouse) | Abcam, Cambridge, UK | ab270993 |
| TIMP1 | Abcam, Cambridge, UK | ab216432 |
| GPX4 | Abcam, Cambridge, UK | ab125066 |
| SCL7A11 | Abcam, Cambridge, UK | ab175186 |
| ACSL4 | Abcam, Cambridge, UK | ab155282 |
| Notch1 | Abcam, Cambridge, UK | ab52627 |
| Notch2 | Abcam, Cambridge, UK | ab307700 |
| Notch3 | Abcam, Cambridge, UK | ab23426 |
| Notch4 | Abcam, Cambridge, UK | ab184742 |
| Jagged1 | Abcam, Cambridge, UK | ab7771 |
| Jagged2 | Abcam, Cambridge, UK | DF7616 |
| Dll1 | Abcam, Cambridge, UK | ab10554 |
| Dll3 | Abcam, Cambridge, UK | ab229902 |
| Dll4 | Abcam, Cambridge, UK | ab183532 |
| Hes1 | Abcam, Cambridge, UK | ab71559 |
| Hes5 | Abcam, Cambridge, UK | ab194111 |
| Hey1 | Abcam, Cambridge, UK | ab235173 |
| Hey2 | Bioss, Woburn, MA, USA | bs-9461R |
| β-actin | Abcam, Cambridge, UK | ab8226 |

| **2.** Antibody for Immunohistochemical staining. | | |
| --- | --- | --- |
| Antibodies | Source | Identifier |
| α-SMA | Abcam, Cambridge, UK | ab124964 |

| **3.** Antibodies for Immunofluorescence Staining. | | |
| --- | --- | --- |
| Antibodies | Source | Identifier |
| α-SMA (human and mouse) | Affinity, Cincinnati, OH, USA | bf9212 |
| Type I clollagen (human) | Abcam, Cambridge, UK | ab138492 |
| Type I clollagen (mouse) | Abcam, Cambridge, UK | ab270993 |
| Jagged1(human and mouse) | Abcam, Cambridge, UK | ab300561 |

| Table S3 Equipment | | |
| --- | --- | --- |
| Equipment | Brand | City, Country |
| Microscope | Zeiss | Jena, Germany |
| ZEISS LSM 880 confocal microscope | Zeiss | Jena, Germany |
| Transmission electron microscope | Hitachi | Tokyo, Japan |
| Microplate Reader | Thermo Fisher Scientific Inc | MA, USA |
| Illumina NovaSeq6000 sequencer | Illumina | San Diego, CA, USA |
| Dual-Luciferase  Reporter Assay System | Promega | Madison, WI, USA |

| **Table S4 Reagents** | | | |
| --- | --- | --- | --- |
| Reagents | Brand | City, Country | Identifier |
| CCl_4_ | Sigma | St Louis, MO, USA | 488488 |
| Olive Oil | Macklin | Shanghai, China | O815210 |
| ALT assay | Nanjing Jiancheng Bioengineering Institute | Nanjing, China | [C009-2-1](http://www.njjcbio.com/products.asp?id=776) |
| AST assay | Nanjing Jiancheng Bioengineering Institute | Nanjing, China | [C010-2-1](http://www.njjcbio.com/products.asp?id=779) |
| TRIzol reagents | Sigma | St Louis, MO, USA | T9424 |
| FBS | Gibco | NY, USA | 10099141 |
| Cell Counting Kit-8 assay | Beyotime | Shanghai, China | C0038 |
| Seq-StarTM poly(A) mRNA Isolation Kit | Arraystar | MD, USA | AS-MB-006-02 |
| KAPA Stranded mRNA-seq Kit | Roche | Pleasanton, CA | 07962169001 |
| EpiQuik m^6^A RNA Methylation Quantitative kit | Epigentek Group Inc. | Farmingdale, NY, USA | P-9005 |
| Bodipy | Invitrogen | California CA, USA | D3922 |
| Iron Assay kit | Abcam | Cambridge, UK | ab83366 |
| HE Staining Kit | Beyotime | Jiangsu, China | C0105 |
| Sirius Red Stain Kit | Abcam | Cambridge, UK | ab150681 |
| Reagents | Brand | City, Country | Identifier |
| Masson Staining Kit | Abcam | Cambridge, UK | ab150669 |
| Polyvinylidene Difluoride Membrane | Thermo Fisher Scientific Inc | MA, USA | ISEQ00010 |
| Puromycin | Sigma | St Louis, MO, USA | [540222](https://www.sigmaaldrich.cn/CN/zh/product/mm/540222) |
| Uranyl Acetate | Hede biotechnology | China | SPI-02624 |
| Lead Citrate | Sigma | St Louis, MO, USA | 15326 |
| Ferrostatin-1 | Selleck Chemicals | Houston, TX, USA | [S7](https://www.sigmaaldrich.cn/CN/zh/product/sigma/sml0583)243 |
| RSL3 | Selleck Chemicals | Houston, TX, USA | S8155 |
| BODIPY-C11 dye | Thermo Fisher Scientific | MA, USA | D3861 |
| MDA Assay Kit | Abcam | Cambridge, UK | ab118970 |
| GSH Assay Kit | Sigma | St Louis, MO, USA | CS0260 |
| ChIP Assay Kit | Genmed | Shanghai, China | GMS30075.1 |
| Formalin Fixative | Sigma | St Louis, MO, USA | Z2902 |
| DMEM | Gibco | NY, USA | 12430054 |
