# Supplementary figures and images for: The IGF2BP3/Notch/Jag1 pathway: A key regulator of hepatic stellate cell ferroptosis in liver fibrosis
Source: Clin Transl Med. 2024 Aug 7;14(8):e1793. doi: 10.1002/ctm2.1793 (PMC11306284; doi:10.1002/ctm2.1793)

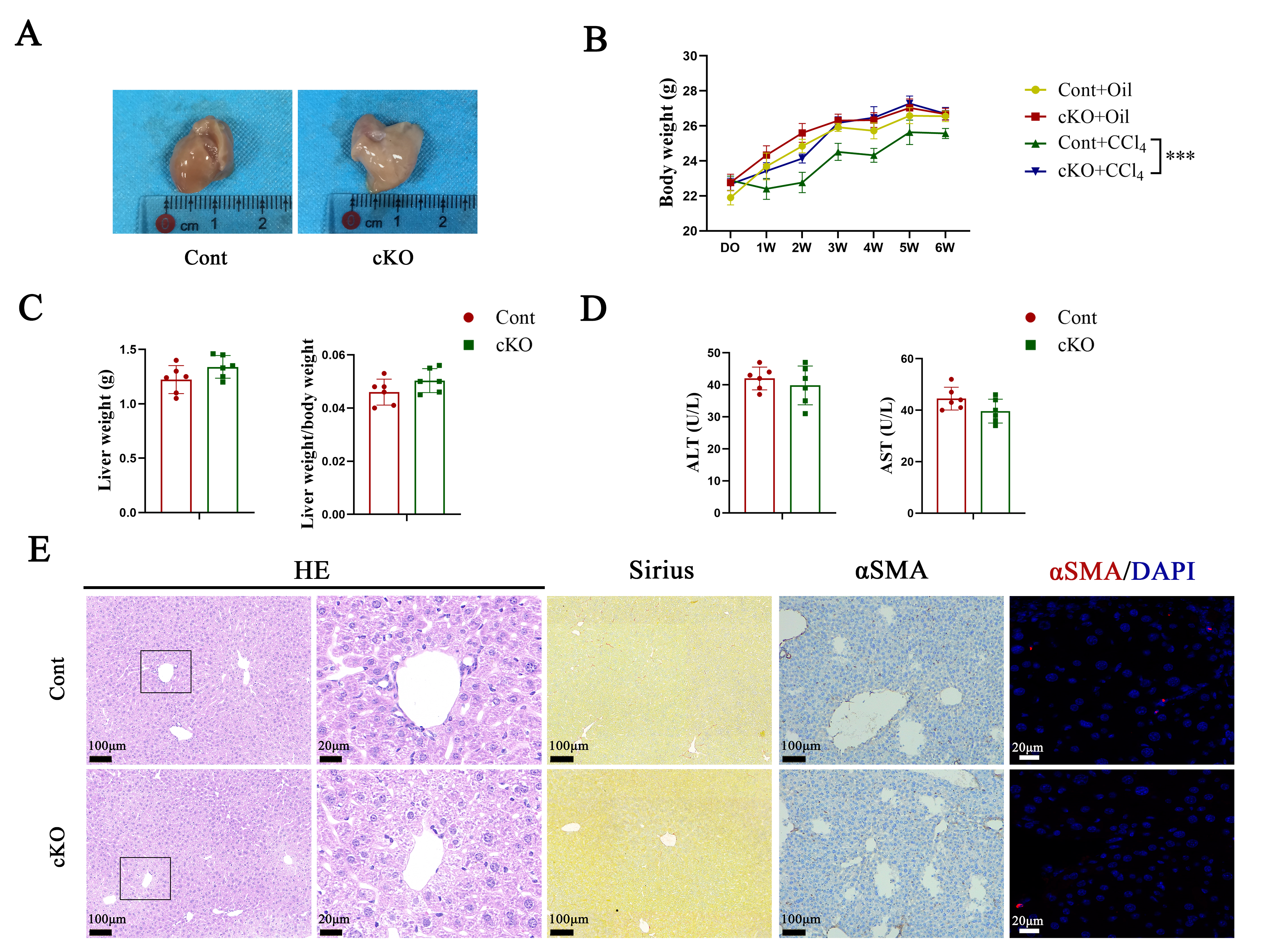

Supplement: Supplementary file 2 — Supporting information [file CTM2-14-e1793-s002.tif]

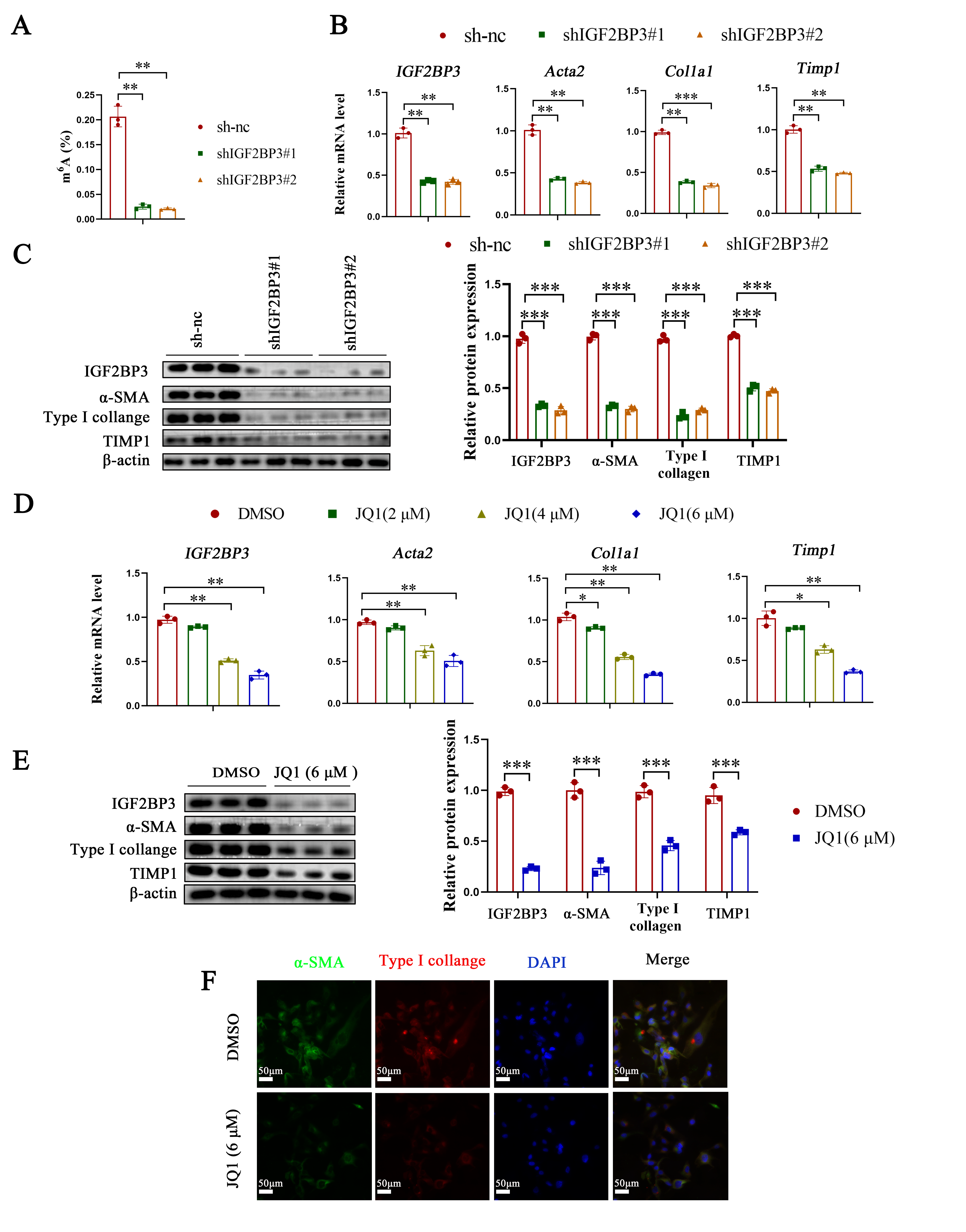

Supplement: Supplementary file 3 — Supporting information [file CTM2-14-e1793-s005.tif]

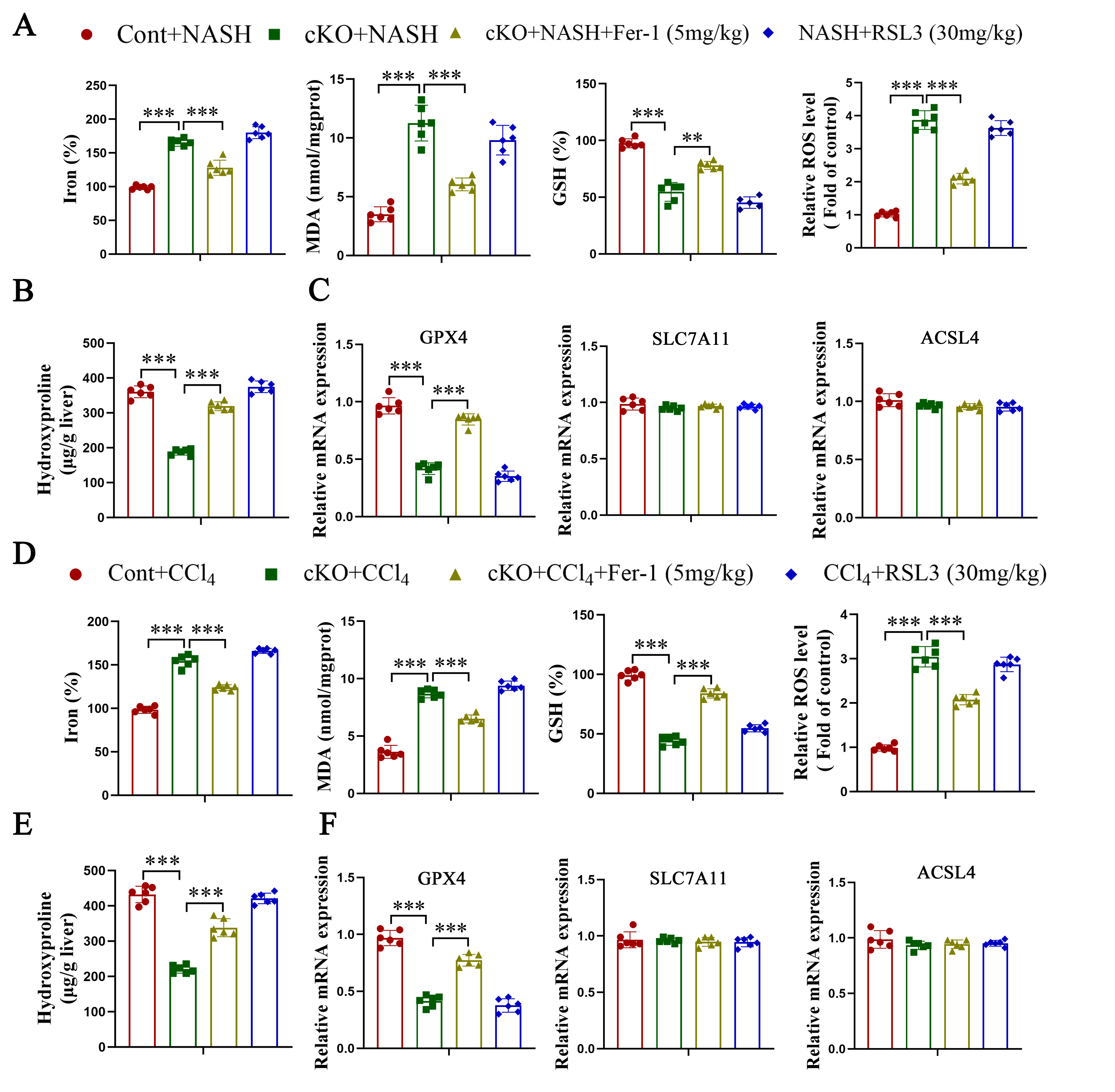

Supplement: Supplementary file 4 — Supporting information [file CTM2-14-e1793-s003.tif]

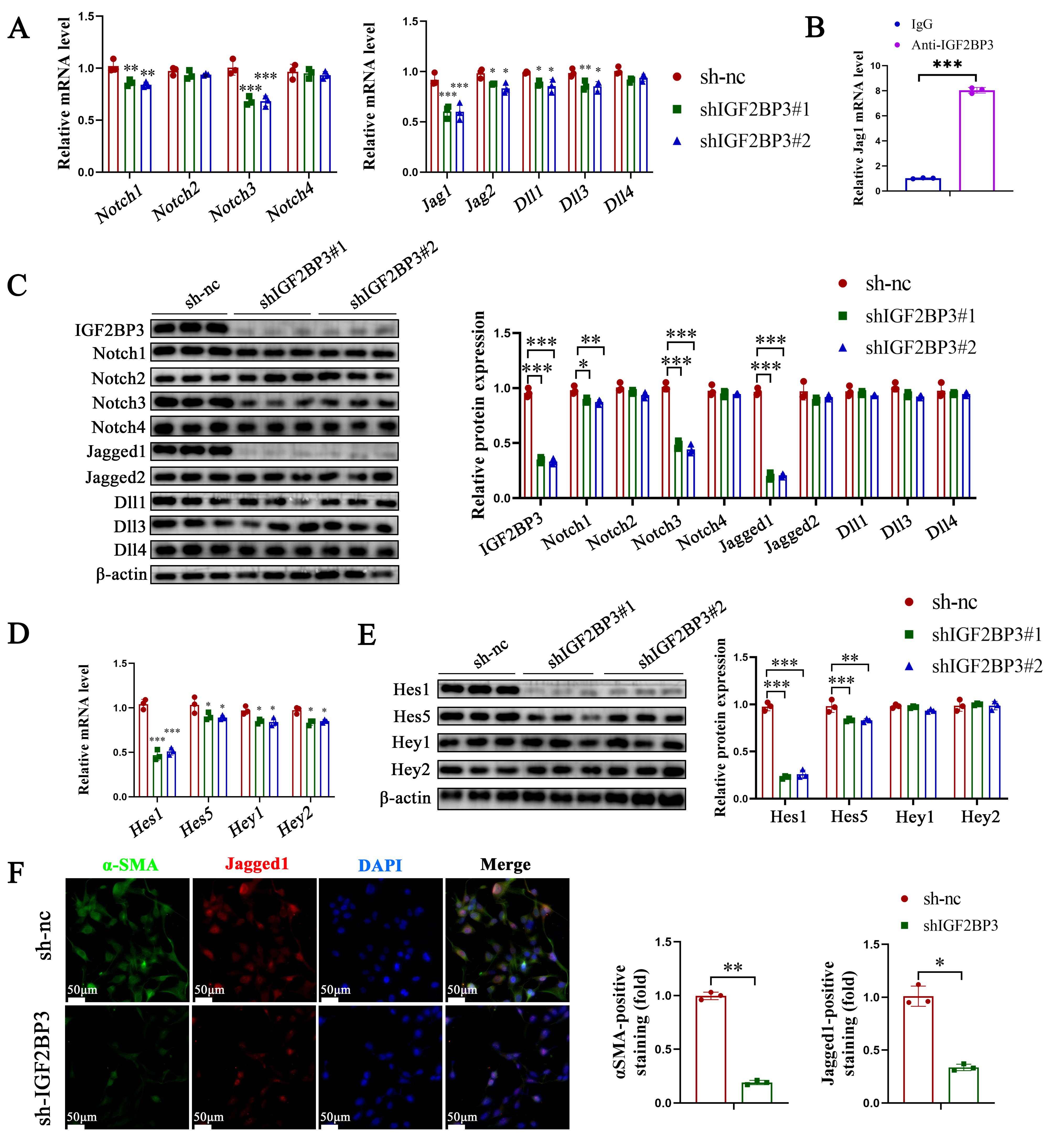

Supplement: Supplementary file 5 — Supporting information [file CTM2-14-e1793-s008.tif]

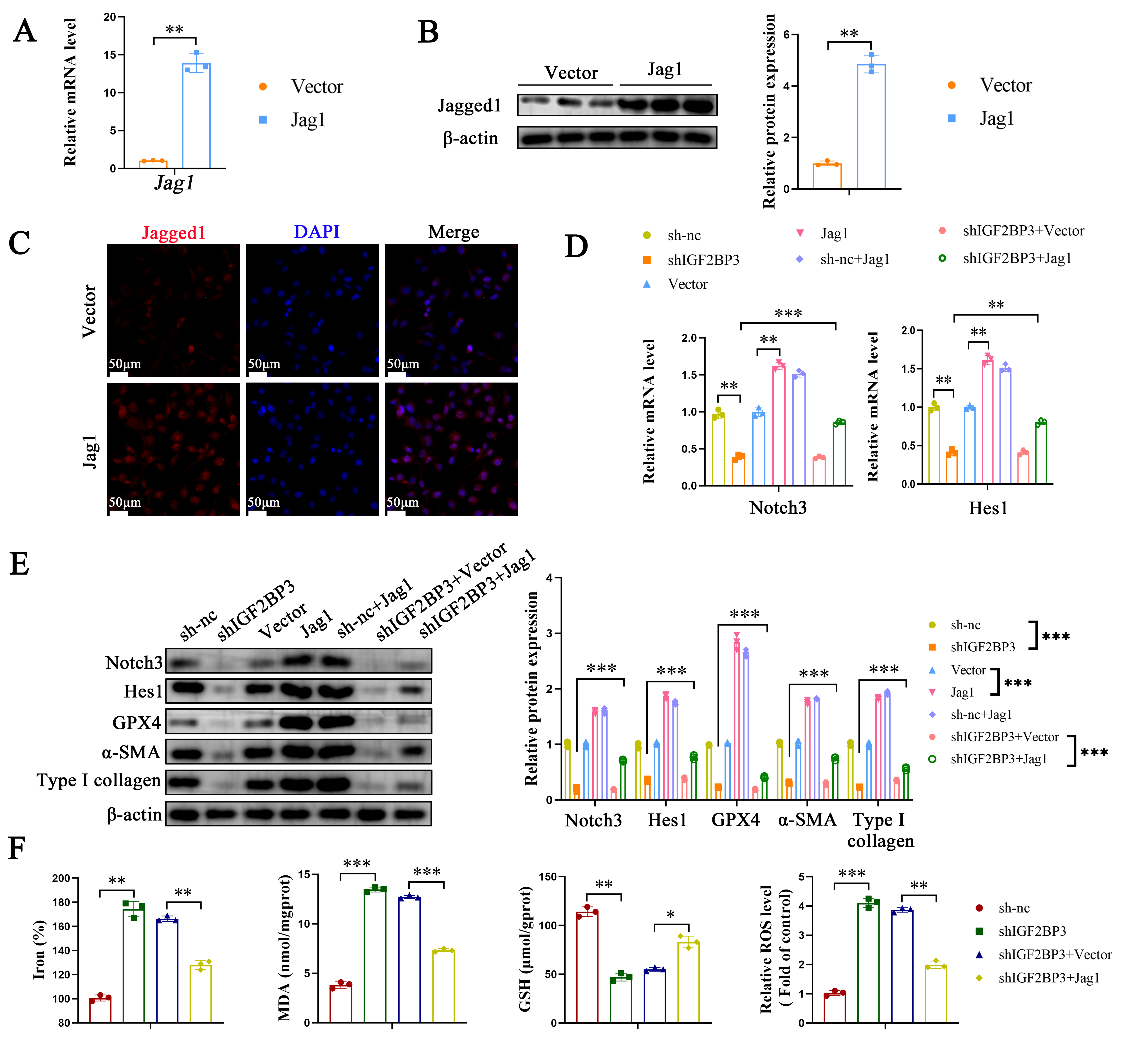

Supplement: Supplementary file 6 — Supporting information [file CTM2-14-e1793-s001.tif]

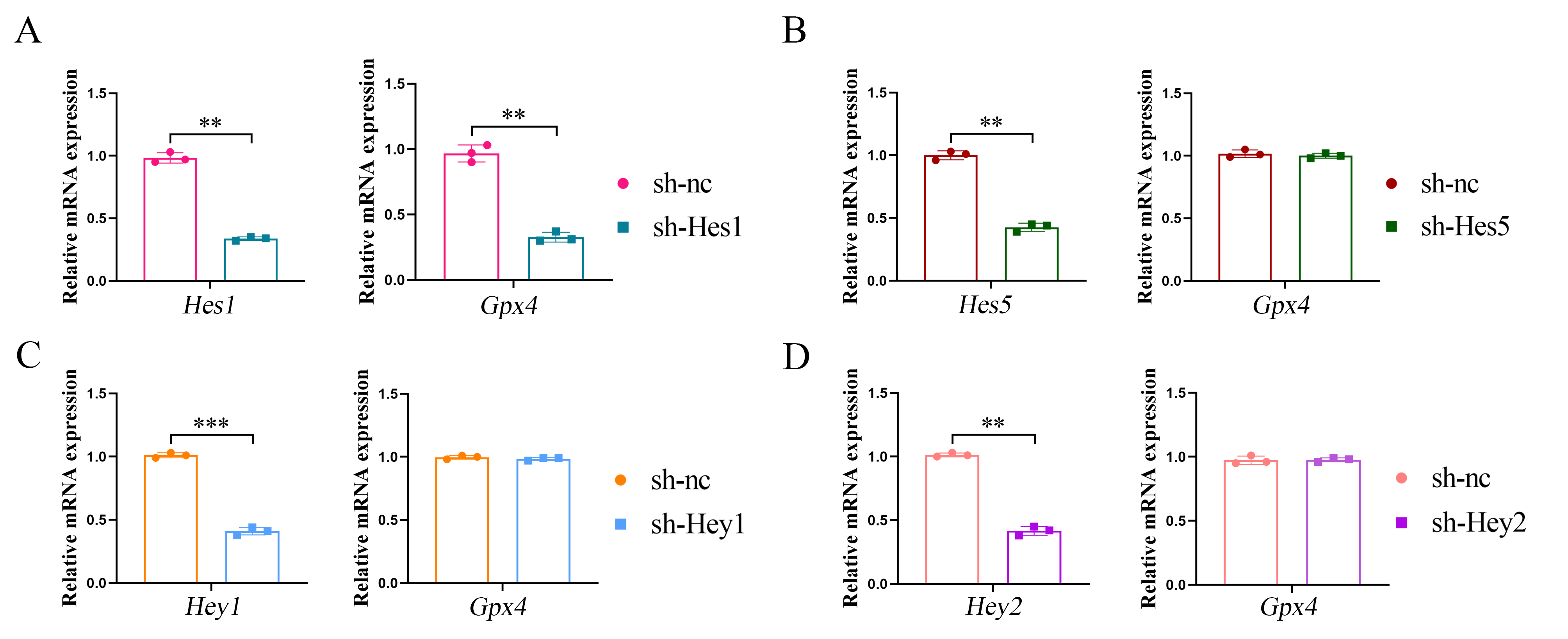

Supplement: Supplementary file 7 — Supporting information [file CTM2-14-e1793-s004.tif]

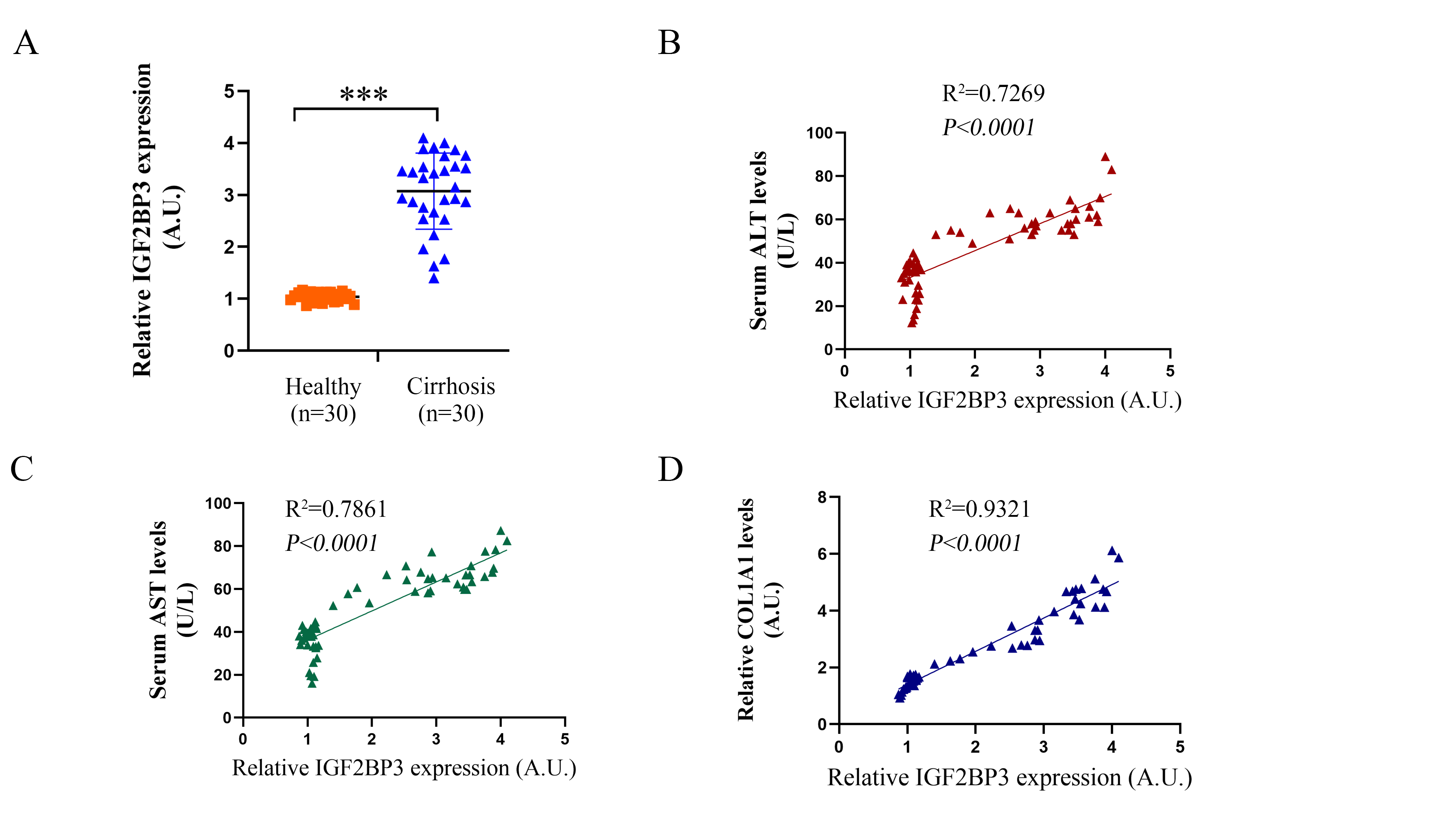

Supplement: Supplementary file 8 — Supporting information [file CTM2-14-e1793-s006.tif]
